# Exploring the impact of maternal factors and dietary habits on human milk oligosaccharide composition in early breastfeeding among Mexican women

**DOI:** 10.1038/s41598-024-63787-1

**Published:** 2024-06-26

**Authors:** Víctor H. Urrutia-Baca, Janet A. Gutiérrez-Uribe, Perla A. Ramos-Parra, Astrid Domínguez-Uscanga, Nora A. Rodriguez-Gutierrez, Karla L. Chavez-Caraza, Ilen Martinez-Cano, Alicia S. Padilla-Garza, Elias G. Ruiz-Villarreal, Francisca Espiricueta-Candelaria, Cristina Chuck-Hernández

**Affiliations:** 1 Tecnologico de Monterrey, Institute for Obesity Research, Ave. Eugenio Garza Sada 2501, 64849 Monterrey, NL Mexico; 2 Tecnologico de Monterrey, Escuela de Ingeniería y Ciencias, Ave. Eugenio Garza Sada 2501, 64849 Monterrey, NL Mexico; 3 Tecnologico de Monterrey, Escuela de Medicina y Ciencias de la Salud, Ave. Ignacio Morones Prieto 3000, 64710 Monterrey, NL Mexico; 4 Facultad de Medicina, Universidad Autónoma de Nuevo León, Ave. Dr. José Eleuterio González 235, 64460 Monterrey, NL Mexico

**Keywords:** Health care, Nutrition

## Abstract

Human milk oligosaccharides (HMOs) promote adequate intestinal microbiota development and favor the immune system's maturation and cognitive development. In addition to non-modifiable factors, HMOs composition can be influenced by other factors like body mass index and eating habits, but the reports are discrepant. The aim of this work was to describe the correlation between maternal factors and HMOs concentration in colostrum in 70 women from northeastern Mexico categorized into women with normal weight and women with overweight or obesity. The absolute concentration of six HMOs were significantly lower in women with overweight or obesity compared to women with normal weight (LNFPI p = 0.0021, 2’-FL p = 0.0304, LNT p = 0.0492, LNnT p = 0.00026, 3’-SL p = 0.0476, 6’-SL p = 0.00041). Another main finding was that the frequency of consumption of food groups such as vegetables, fruits and meats was positively correlated to specific HMOs (Poblano chili and 2’-FL; *r*_*s*_ = 0.702, p = 0.0012; Orange or tangerine and 3-FL; *r*_*s*_ = 0.428, p = 0.0022; Chicken and 2'-FL; *r*_*s*_ = 0.615, p = 0.0039). This study contributes to the elucidation of how maternal factors influence the composition of HMOs and opens possibilities for future research aimed at mitigating overweight or obesity, consequently improving the quality of human milk.

## Introduction

Health authorities around the world have recognized the nutritional, physiological, and protective benefits of breastfeeding, such as the tendency to reduce the risk of type 2 diabetes, obesity, allergies, celiac disease, necrotizing enterocolitis (NEC), gastrointestinal tract infections, and some cancers. HMOs are multifunctional glycans naturally present in human milk and are particularly interesting for their quantity and structural diversity^1^. So far, more than 200 individual molecular species of HMOs and more than 100 structures have been reported^2^. HMOs contain glucose (Glc), galactose (Gal), *N*-acetylglucosamine (GlcNac), fucose (Fuc), and *N*-acetylneuraminic acid (Neu5Ac) and are formed by a reducing lactose core that can be extended enzymatically by lacto-*N*-biose (Galβ-1,3-GlcNAc, type 1 LacNAc) or N-acetyllactosamine (Gal-β1,4-GlcNAc, type 2 LacNAc) motifs. These structures can be further decorated by the addition of Fuc residues in α1,2-, α1,3-, and α1,4-linkages or Neu5Ac residues in α2,3- and α2,6-linkages.

Since oligosaccharide chains can be either fucosylated or sialylated, they are usually classified into three main categories: neutral non-fucosylated, neutral fucosylated, and HMOs containing sialic acid. HMOs promote the colonization of beneficial microbes in the infant's gut, such as beneficial Bifidobacterium species, preventing the growth of other harmful bacteria and contributing to the development of the immune system^3^. HMOs have been shown to have anti-bacterial, anti-viral, and anti-inflammatory effects and have proven beneficial toward the protection from NEC^4,5^. The benefits of HMOs may extend beyond infancy to the development of cognitive functions, making them the focus of intense current scientific research.

In addition to genetic factors, environmental factors such as geographical location, season of delivery, and maternal diet and maternal characteristics such as age, ethnicity, parity, mode of delivery, and maternal nutritional status affects HMOs concentration and composition^6–9^. Body mass index (BMI) at the beginning of pregnancy has been related to differences in the concentration of HMOs. Some studies have reported that overweight and obesity may positively or negatively correlate with the concentration of specific oligosaccharides^7,8,10–12^. McGuire et al.^7^, in 410 breastfeeding women from an international cohort (Spanish, Swedish, Peruvian, North American, Ethiopian, Gambian, Ghanaian, and Kenyan) found that maternal weight and BMI were positively associated with 2′-FL and fucosyl-lacto-*N*-hexaose (FLNH); maternal weight was positively correlated with lacto-*N*-fucopentaose III (LNFP III) and difucosyl-lacto-*N*-tetraose (DFLNT). Conversely, maternal weight and BMI were inversely correlated with LNnT and disialyl-lacto-N-tetraose (DSLNT). Azad et al.^8^, observed that lacto-*N*-hexaose (LNH) was lower in women with overweight compared to women with normal weight while DFLNT was higher in women with obesity. Isganaitis et al.^12^, found that three HMOs along with other seven metabolites concentrations were significantly different in the milk of one month infant mothers from Oklahoma (USA) with overweight or obesity compared to normal weight at one month after delivery; particularly Lacto-*N*-fucopentaose-I (LNFP-I) and 2’-FL contents were reduced by 62% and 38%, respectively, while Lacto-N-fucopentaose-II (LNFP-II) or Lacto-*N*-fucopentaose-III (LNFP-III) increased by 65%^12^.

In another study in a European cohort, women with overweight had significantly higher concentrations of 3’-SL, 6’-galactosyl-lactose (6’-GL) and DSLNT at day 2; 6’-SL at day 17; and lacto-N-fucopentaose-V (LNFP-V) at day 90 and 120 than women with normal weight. In addition, lower concentrations of LNnT (at day 2 postpartum), lacto-N-tetraose (LNT; at day 30 and 90 postpartum), and LNFP-V (at day 60 postpartum) were observed in women with overweight or obesity compared to women with normal weight^10^. Ferreira et al.^11^, in 101 Brazilian mothers reported a direct correlation between LNnT and prepregnancy weight and BMI and an inverse correlation with 3-fucosyllactose (3-FL), LNFP-III and DFLNH at 2–8 days. However, a study reported that prepregnancy BMI was not associated with the concentration of HMOs in Hispanic mothers living in Los Angeles, CA, USA^13^.

To understand the influence of obesity on the breast milk composition, Saben et al.^14^, were the first to evaluate the relationship between obesity-associated maternal factors (as hyperglycemia, hyperinsulinemia, and insulin resistance) and HMOs in 136 US mothers. They observed a negative association between third trimester fasting plasma glucose and insulin with total HMO-bound sialic acid and concentrations of the sialylated HMOs 3’-SL and DSLNT in non-secretors (women who are unable to produce 2’-FL and LNFPI in breast milk) at two months postpartum. In secretors, difucosyllactose and LNFP-II concentrations increased, and sialyllacto-*N*-tetraose c (LSTc) and sialyllacto-*N*-tetraose b (LSTb) decreased as insulin sensitivity increased^14^.

A recent study discovered significant associations with changes in maternal food intake during lactation. Higher cheese, egg, fruits and vegetables consumption has been positively correlated with HMO concentrations in secretory mothers (for example, 2'-FL and LNT + LNnT), while fish consumption has been negatively associated with LNFP-V^15^. Previous studies have also demonstrated the association of food consumption with HMOs composition with contradictory results between them^8,15–17^.

Undoubtedly, the discrepancy between the different reports invites new research on the influence of modifiable factors on HMOs. The aim of the present study was to describe the correlation between maternal factors and HMOs concentrations at the beginning of breastfeeding.

## Results

### Participant demographics, medical history, and anthropometrics: mother–child pairs

In the present study, 70 mothers and their newborn children were recruited. Of the participating mothers, 94.3% were secretors (HMOs production status). They had an average age of 23.0 ± 5.2 years at delivery (Table 1). Regarding their demographics, 80% resided in the Monterrey N.L. Mexico metropolitan area, 60% had middle school as their highest level of completed education, and 90% of the mothers were employed in various unspecified occupations. Medically, 10% had a history of high blood pressure and 5.7% of cholesterolemia or dyslipidemia; none had a history of kidney disease. Cholesterolemia or dyslipidemia was found to be associated with overweight or obesity in mothers (p = 0.05); the four women who had this medical history were in the overweight or obesity group, as detailed in Table 1.

**Table 1:** Comparative characteristics of Mexican mother-infant pairs: women with normal weight *vs*. women with overweight or obesity.

	Total (n = 70)		Women with normal weight (n = 36)		Women with overweight or obesity (n = 34)		P-value
Women n (%) or mean ± SD							
Secretor status							
Yes	66	(94.3)	35	(50.0)	31	(44.3)	0.276^a^
No	4	(5.7)	1	(1.4)	3	(4.3)	
Age at delivery (years)	23.0	± 5.2	22.6	± 5.2	23.5	± 5.2	0.448^b^
Residence							
Metropolitan area	56	(80.0)	29	(41.4)	27	(38.6)	0.905^a^
Non-metropolitan area	14	(20.0)	7	(10.0)	7	(10.0)	
Maximum level of studies							
Elementary school	15	(21.4)	5	(7.1)	10	(14.3)	0.257^a^
Middle school	42	(60.0)	22	(31.4)	20	(28.6)	
High school	12	(17.1)	8	(11.4)	4	(5.7)	
Bachelor	1	(1.4)	1	(1.4)	0	(.0)	
Vocational school	0	(.0)	0	(.0)	0	(.0)	
Occupation							
Merchants, sales employees, and sales agents	4	(5.7)	3	(4.3)	1	(1.4)	0.524^a^
Other workers with unspecified occupations	63	(90.0)	31	(44.3)	32	(45.7)	
Workers in domestic services	3	(4.3)	2	(2.9)	1	(1.4)	
High blood pressure							
Yes	7	(10.0)	3	(4.3)	4	(5.7)	0.632^a^
No	63	(90.0)	33	(47.1)	30	(42.9)	
Kidney disease							
Yes	0	(.0)	0	(.0)	0	(.0)	na^c^
No	70	(100.0)	36	(51.4)	34	(48.6)	
Cholesterolemia or dyslipidemia							
Yes	4	(5.7)	0	(.0)	4	(5.7)	**0.05**^**a**^
No	66	(94.3)	36	(51.4)	30	(42.9)	
According to the type of birth at the beginning							
Induced	23	(32.9)	9	(12.9)	14	(20.0)	0.15^a^
Spontaneous	47	(67.1)	27	(38.6)	20	(28.6)	
According to your type of birth completion							
Eutocic	54	(77.1)	31	(44.3)	23	(32.9)	0.089^a^
Dystocytic	16	(22.9)	5	(7.1)	11	(15.7)	
Previous births (parity)	1.3	± 1.4	1.2	± 1.4	1.4	± 1.4	0.755^b^
Number of children in current delivery	1	± 0	1	± 0	1	± 0	na^c^
Diastolic blood pressure (mm Hg)	69.4	± 11.1	69.4	± 12.3	69.5	± 9.9	0.967^b^
Systolic blood pressure (mm Hg)	113.0	± 11.2	109.7	± 11.8	116.4	± 9.4	**0.01**^b^
Prepregnancy BMI (kg m^−2^)	24.5	± 5.9	20.9	± 2.1	28.2	± 6.4	** < 0.001**^b^
BMI at delivery (kg m^−2^)	28.3	± 5.7	24.4	± 2.0	32.6	± 5.4	** < 0.001**^b^
Weight gain (kg)	9.8	± 7.1	8.4	± 4.2	11.3	± 9.0	0.085^b^
Infants n (%) or mean ± SD							
Birth weight (kg)	3.2	± 0.4	3.1	± 0.4	3.3	± 0.4	**0.024**^b^
Birth height (cm)	49.2	± 2.1	49.4	± 2.2	49.1	± 2	0.588^b^
Birth weight evaluation							
Risk of malnutrition	18	(25.7)	13	(18.6)	5	(7.1)	0.089^a^
Normal weight	48	(68.6)	22	(31.4)	26	(37.1)	
Overweight	4	(5.7)	1	(1.4)	3	(4.3)	
Birth height evaluation							
Risk of short height	13	(18.6)	6	(8.6)	7	(10.0)	0.673^a^
Normal height	57	(81.4)	30	(42.9)	27	(38.6)	
Birth head circumference (cm)	33.6	± 1.3	33.5	± 1.3	33.8	± 1.2	0.44^b^
Sex							
Female	42	(60.0)	20	(28.6)	22	(31.4)	0.435^a^
Male	28	(40.0)	16	(22.9)	12	(17.1)	
Congenital anomalies							
Yes	3	(4.3)	2	(2.9)	1	(1.4)	0.589^a^
No	67	(95.7)	34	(48.6)	33	(47.1)	
APGAR score							
0–3 (severe depression)	1	(1.4)	1	(1.4)	0	(.0)	0.328^a^
7–10 (normal)	69	(98.6)	35	(50.0)	34	(48.6)	
Silverman-Andersen score							
0 without RD	66	(94.3)	35	(50.0)	31	(44.3)	0.468^a^
1–3 RD mild	3	(4.3)	1	(1.4)	2	(2.9)	
7–10 RD severe	1	(1.4)	0	(.0)	1	(1.4)	
Hearing screening							
Negative	14	(20.0)	10	(14.3)	4	(5.7)	0.058^a^
Positive	32	(45.7)	18	(25.7)	14	(20.0)	
Not specified	24	(34.3)	8	(11.4)	16	(22.9)	

^a^Chi-squared and Fisher's exact test.

^b^Student’s t-test.

^c^Not applied.

Significant values are in bold.

Regarding the data associated with pregnancy and childbirth, 67.1% of births were spontaneous at the beginning, and 77.1% of the deliveries were eutocic. According to their conclusion, each delivery resulted in the birth of a single child. The average number of previous births compared to the current was 1.3 ± 1.4 children. Based on the anthropometric data in all participants, the prepregnancy BMI was 24.5 ± 5.9 kg m^-2^, and the BMI at delivery was 28.3 ± 5.7 kg m^-2^ with a total weight gain of 9.8 ± 7.1 kg. The prepregnancy BMI and BMI at delivery were significantly higher in women with overweight or obesity compared to the women with normal weight, as expected (Table 1). Furthermore, the diastolic and systolic blood pressures in all participants were 69.4 ± 11.1 mm Hg and 113.0 ± 11.2 mm Hg, respectively. Systolic blood pressure was significantly higher (p = 0.01) in women with overweight or obesity (116.4 ± 9.4 mm Hg) compared to women with normal weight (109.7 ± 11.8 mm Hg). However, 4/36 (11.1%) and 3/34 (8.8%) showed a diastolic blood pressure above 80 mm Hg in women with normal weight and women with overweight or obesity, respectively. On the other hand, 6/36 (16.6%) and 9/34 (26.5%) showed a systolic blood pressure above 120 mm Hg in women with normal weight and women with overweight or obesity, respectively. Regarding to another maternal factors such as age at delivery, residence, maximum level of studies, occupation, high blood pressure medical history, mode of delivery, parity, diastolic blood pressure and weight gain, no differences were found between the groups (women with overweight or obesity *vs.* women with normal weight; p > 0.05), as shown in Table 1.

Data were obtained from all participating mothers' newborns, 60% were female, and 95.7% did not show congenital anomalies. The weight and height at birth were 3.2 ± 0.4 kg and 49.2 ± 2.1 cm, respectively. Of newborns, 68.6% were normal weight, and 81.4% were normal height. The weight of the newborns was significantly higher in women with overweight or obesity compared to women with normal weight (p = 0.024). In addition, the average birth head circumference was 33.6 ± 1.3 cm. Regarding neonatal screening, APGAR and Silverman-Andersen scores were normal in most newborns, and hearing screening was positive (a positive result indicates correct hearing at birth) in 45.7% (Table 1). Regarding birth height, birth head circumference, sex, congenital anomalies, APGAR and Silverman-Andersen scores, and hearing screening, no differences were found between the groups (p > 0.05).

### HMOs profiling and individual concentrations between the study groups

**Figure 1:**
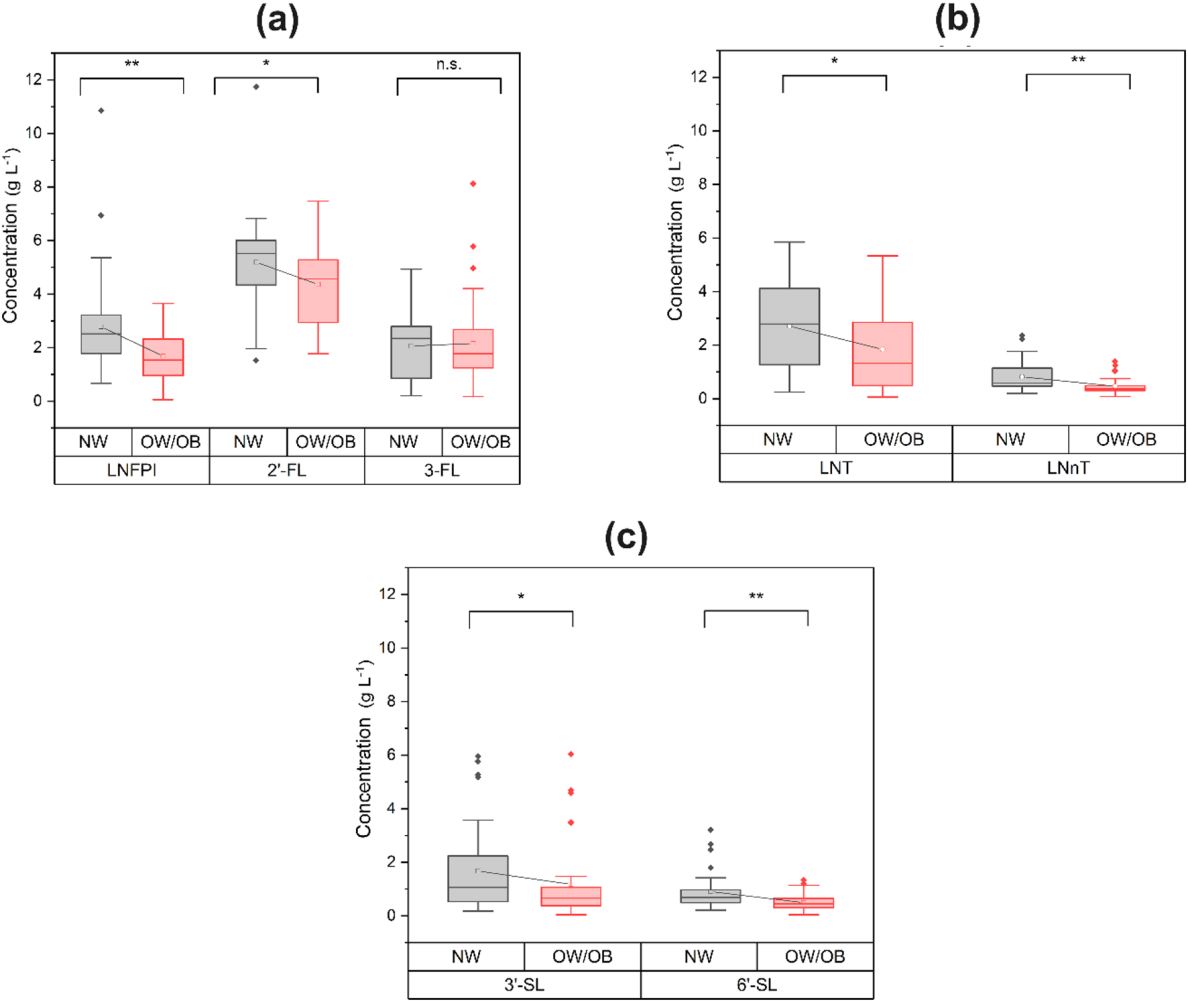
Comparison of HMO concentrations in colostrum samples from Mexican mothers with normal weight and overweight/obesity. (**a**) Neutral fucosylated, (**b**) neutral no fucosylated and (**c**) acidic. The data is displayed in a grouped boxplot with connect mean line and the p values were obtained using the non-parametric Mann–Whitney U test. Significance levels are denoted as * for p < 0.05, ** for p < 0.01 and *** for p < 0.001; *n.s.* not significant. *NW* women with normal weight, *OW/OB* women with overweight/obesity, *2’-FL* 2’-fucosyllactose, *3’-SL* 3’-sialyllactose, *6’-SL* 6’-sialyllactose, *3-FL* 3-fucosyllactose, *LNFPI* lacto-*N*-fucopentaose I, *LNnT* lacto-*N*-neotetraose, *LNT* lacto-*N*-tetraose.

The total HMOs content was significantly lower in the group of women with overweight or obesity with 11.7 ± 3.4 g L^−1^ compared to women with normal weight with 15.9 ± 4.7 g L^−1^ (p < 0.001). These concentrations were based on the sum of 2'-FL, 3-FL, LNT, LNnT, LNFPI, 3'-SL, and 6'-SL, as the most representative HMOs (about 90%) in breast milk (Fig. 1 and Supplementary Table 2). In all participants, about 63% of the total HMOs were fucosylated neutrals (Fig. 1a), followed by 21.9% non-fucosylated neutrals (Fig. 1b) and 15.1% acidic (Fig. 1c).

There was a significant decrease in the concentration of six of the seven HMOs in women with overweight or obesity compared to women with normal weight. For the neutral fucosylated HMOs, the concentrations of 2’-FL were 5.2 ± 1.8 g L^−1^, and LNFPI were 2.7 ± 1.9 g L^−1^ in women with normal weight compared to women with overweight or obesity with 4.4 ± 1.4 g L^−1^ and 1.7 ± 0.9 g L^−1^, respectively (Fig. 1a). In addition, the acidic HMOs 3’-SL and 6’-SL showed concentrations of 1.9 ± 1.3 g L^−1^ and 1.1 ± 0.5 g L^−1^ in women with normal weight compared to women with overweight or obesity with 1.2 ± 1.5 g L^−1^ and 0.5 ± 0.3 g L^−1^, respectively (Fig. 1c). In the case of non-fucosylated neutral HMOs, the concentrations of LNT and LNnT were 2.7 ± 1.8 g L^−1^ and 0.8 ± 0.5 g L^−1^ in women with normal weight compared to women with overweight or obesity with 1.8 ± 1.5 g L^−1^ and 0.5 ± 0.3 g L^−1^, respectively (Fig. 1b).

### Correlations between HMOs concentrations and maternal factors

**Figure 2:**
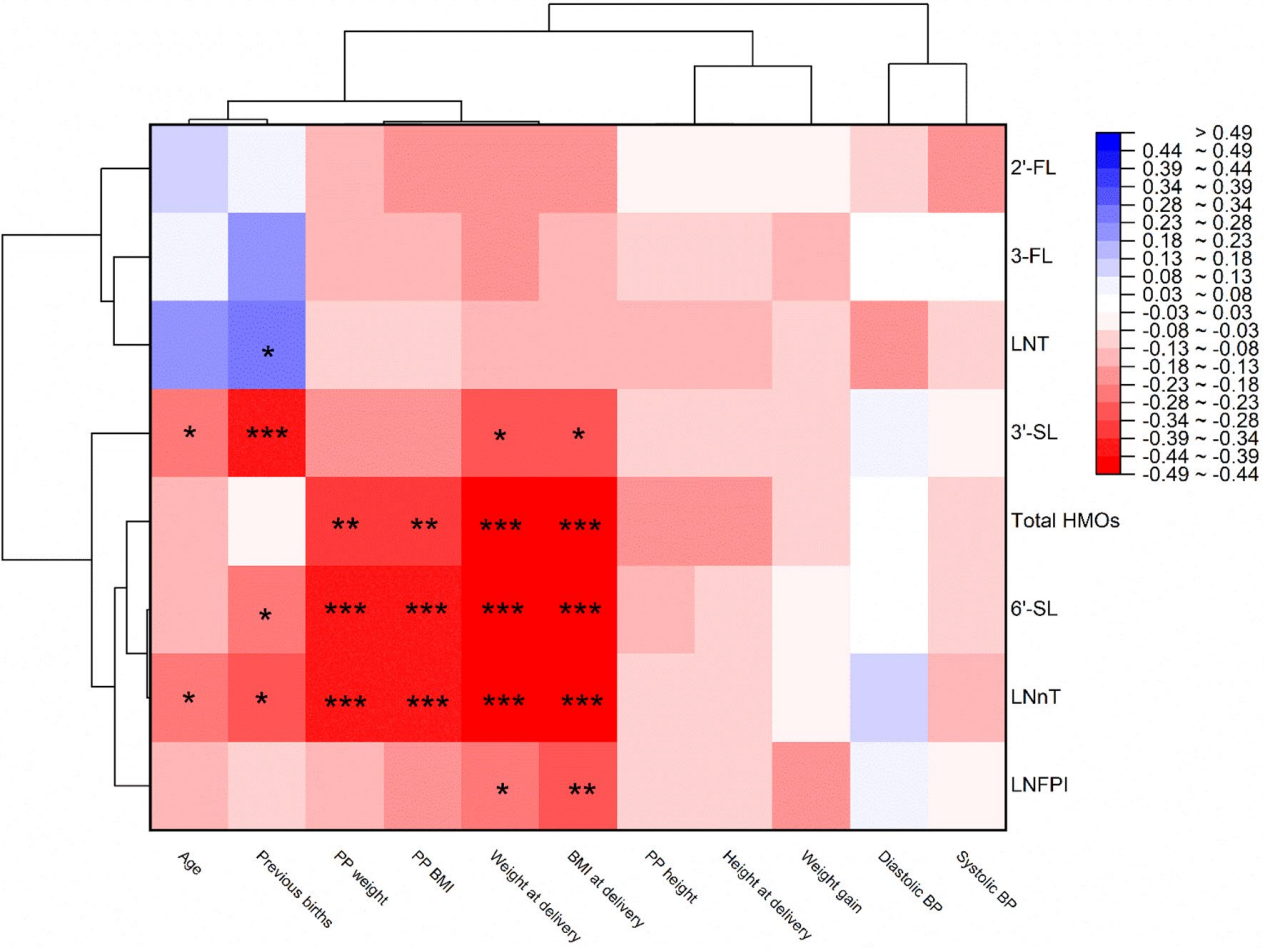
Heatmap and dendrogram of Spearman’s correlation coefficients between HMOs concentrations and factors including age, anthropometrics, and previous births. Red squares indicate negative correlations, while blue squares indicate positive correlations. Significance levels are denoted as * for p < 0.05, ** for p < 0.01 and *** for p < 0.001. *PP* prepregnancy, *BP* blood pressure.

Three main horizontal clusters were observed among maternal factors in the hierarchical analysis (Fig. 2). The first was composed of age, parity, prepregnancy weight and BMI, and weight and BMI at delivery, while the second was composed of prepregnancy height, height at delivery and weight gain, and the third group composed by diastolic and systolic blood pressure. Only the factors grouped in the first cluster showed correlations with HMOs. HMOs were arranged in two main vertical clusters; the first formed by LNT, 3-FL and 2'-FL while the second by LNFPI, LNnT, 6'-SL, total HMOs and 3'-SL. Negative correlations associated with age, previous births, prepregnancy weight, prepregnancy BMI, weight at delivery, and BMI at delivery were observed with HMOs concentrations at the beginning of lactation.

Age showed a weak negative correlation with LNnT (*r*_*s*_ = −0.254; p < 0.05) and 3’-SL (*r*_*s*_ = −0.242; p < 0.05). The number of previous births showed a weak negative correlation with LNnT (*r*_*s*_ = −0.295; p < 0.05), 3’-SL (*r*_*s*_ = −0.397; p < 0.001), and 6’-SL (*r*_*s*_ = −0.268; p < 0.05), but a weak positive correlation with LNT (*r*_*s*_ = 0.266; p < 0.05), as shown in Fig. 2.

Pre-pregnancy weight and BMI were weak negatively correlated with total HMOs (*r*_*s*_ = −0.349 and ρ = −0.347; p < 0.01 both), 6’-SL (pre-pregnancy weight: *r*_*s*_ = −0.391; p < 0.001) and moderate negatively correlated 6’-SL (pre-pregnancy: *r*_*s*_ = −0.407; p < 0.001) and LNnT (*r*_*s*_ = −0.407 and *r*_*s*_ = −0.426; p < 0.001 both). In addition, weight and BMI at delivery were moderate negatively correlated with total HMOs (*r*_*s*_ = −0.444 and *r*_*s*_ = −0.451; p < 0.001 both), 6'-SL (ρ = −0.462 and ρ = −0.487; p < 0.001 both) and LNnT (ρ = −0.458 and ρ = −0.491; p < 0.001 both), and weak negatively correlated with 3’-SL (*r*_*s*_ = −0.298 and *r*_*s*_ = −0.302; p < 0.05 both) and LNFPI (*r*_*s*_ = −0.284 and *r*_*s*_ = −0.312; p < 0.05 and p < 0.01), as shown in Fig. 2.

### Correlations between HMOs and frequency of food group consumption

The foods with the highest total weekly consumption frequencies of each food group were whole milk (7.4 ± 6.1), orange or tangerine (7.4 ± 10.0), onion (10.3 ± 9.2), egg-warm or boiled (7.0 + 8.1), fresh fish (2.3 ± 3.0), beans prepared at home-cooked (5.8 ± 6.0), breakfast cereal: fruit flavor—froot loops (8.5 ± 7.8), corn atole—with milk (3.3 ± 5.7), water (24.0 ± 12.4), pasta soup—broth (3.4 ± 4.2), Tortilla (purchased) or factory-made tortilla (29.0 ± 23.3), and salt or seasoning with salt added to the foods (14.1 ± 8.5). In addition, some differences in the total weekly consumption frequencies of specific foods between the study groups were observed, as shown in Supplementary Table 3.

In the dairy foods group, a strong and very strong positive correlation was only observed between the natural drinkable yogurt consumption and two HMOs: LNnT (*r*_*s*_ = 0.756) and 6’-SL (*r*_*s*_ = 0.850). On the other hand, strong negative correlations of the natural whole yogurt (*r*_*s*_ = −0.624) and whole yogurt with fruits (*r*_*s*_ = −0.647) with LNT concentrations were observed. Panela or fresh or cottage cheese consumption was moderate negatively associated with the concentration of acidic HMOs 3-SL (*r*_*s*_ = −0.421) and 6’-SL (*r*_*s*_ = −0.404). In addition, semi or ripened cheese consumption was weak negatively correlated to LNnT (*r*_*s*_ = −0.323) and total HMOs (*r*_*s*_ = −0.317) concentrations, and moderate negatively correlated to 6’-SL (*r*_*s*_ = −0.443), as shown in Table 2.

**Table 2:** Spearman correlations of HMOs concentrations with total weekly frequency of food group consumption.

Food groups		Spearman's correlation coefficient (*r*_*s*_)^a^							
		LNFPI	LNnT	LNT	3'-SL	6'-SL	3-FL	2'-FL	Total HMOs^b^
Dairy foods	Natural whole yogurt			−0.624					
	Whole yogurt with fruits			−0.547					
	Natural drinkable yogurt		0.756			0.850			
	Panela, fresh or cottage cheese				−0.421	−0.404			
	Semi or ripened cheeses (Chihuahua, Gouda, Manchego)		−0.323			−0.443			−0.317
Fruits	Orange or tangerine	0.294					0.428		
	Apple or pear		0.310		0.365	0.411			0.285
	Guava			−0.551					
	Mango		0.362		0.353	0.358			
	Strawberries	0.322	0.316			0.347			0.326
	Grapes						0.307		
	Crystallized or dried fruits						0.748		
Vegetables	Green leaves		0.452						
	Carrot						0.298		
	Lettuce	0.300	0.292		0.279	0.312			0.264
	Nopales	0.316	0.350		0.294	0.351			
	Cucumber						0.370		
	Avocado	0.366	0.381						0.303
	Poblano chili		0.476			0.604		0.702	0.466
	Packaged vegetables	−0.498							
Meats, sausages, and eggs	Pork meat	0.391						0.432	0.382
	Beef								0.269
	Pork, turkey or mixed sausage, pork or turkey ham or mortadella						0.256		
	Chicken (1 piece of wing, 2 pieces of leg)							0.615	
	Egg (1 piece fried, fried or scrambled egg)		0.311						
Fish	Tuna and sardine		0.374						
	Some seafood			0.453					
Legumes	Beans prepared at home (cooked)						0.366		
	Beans prepared at home (refried)						0.597		
Cereals and tubers	Whole grain bread						0.515		
	Sweet bread (except donuts and churros)						0.342		
	Potatoes: half fried piece or half a potato pancake						−0.389		
	Breakfast cereal: fruit flavor (froot loops)						−0.912		
Corn food	Appetizers with vegetables such as sopes, quesadillas, tlacoyos, gorditas and enchiladas (not tacos; without frying)					−0.660			
	Appetizers with vegetables such as sopes, quesadillas, tlacoyos, gorditas and enchiladas (not tacos; fried)						0.509		
Beverages	Natural fruit waters with sugar						0.708		0.664
	Unsweetened industrialized flavored beverages (including diet ones such as Clight, Be-light, etc.)					0.673			
	Fruit nectars or fruit pulp industrialized with sugar (boing, jumex)		0.405		0.443	0.328			
Soups and pasta	Pasta soup (broth)				0.369				
Tortilla	Corn flour tortilla (homemade)		0.712						
Miscellaneous	Dried chili, for example in sauces, tacos, stews (ground or whole)	0.7127					0.81742		
	Soy sauce, Worcestershire sauce, or liquid food seasonings				0.84515				

^a^Only Spearman's correlation coefficient values that had a significant correlation (p < 0.05) are shown. Spearman's correlation coefficient > 0 indicate a positive correlation and < 0 indicate a negative correlation.

^b^The sum is based on 2'-FL, 3-FL, LNT, LNnT, LNFPI, 3'-SL and 6'-SL.

In the fruit group, we found that the consumption of seven different types of fruits was associated with changes in the concentration of HMOs. Guava consumption showed a moderate negative correlation with the level of LNT (*r*_*s*_ = −0.551), whereas from weak to strong positive correlations were: (1) orange or tangerine with LNFPI and 3-FL; (2) apple or pear with LNnT, 3’-SL, 6’-SL, and total HMOs; (3) mango with LNnT, 3’-SL and 6’-SL; (4) strawberries with LNFPI, LNnT, 6’-SL and total HMOs; (5) grapes with 3-FL; and (6) crystallized or dried fruits with 3-FL (Table 2).

Results like those previously described were observed in the vegetable group, seven different vegetables showed positive correlations with the concentrations of specific HMOs. The consumption of frozen vegetables moderate negatively correlated with the LNFPI concentrations (*r*_*s*_ = −0.498). From weak to strong positive correlations are described as follows: (1) green leaves with LNnT; (2) carrot with 3-FL; (3) lettuce with up to four HMOs (LNFPI, LNnT, 3’-SL, and 6’-SL) and total HMOs; (4) nopales (cactus) with up to four HMOs (LNFPI, LNnT, 3’-SL, and 6’-SL); (5) cucumber with 3-FL; (6) avocado with LNFPI and LNnT; and (7) poblano chili with LNnT, 6’-SL, 3-FL and total HMOs.

The consumption of beef, pork, turkey or mixed sausage, pork or turkey ham or mortadella and egg were weak positively associated with the concentrations of total HMOs (*r*_*s*_ = 0.269), 3-FL (*r*_*s*_ = 0.256), and LNnT (*r*_*s*_ = 0.311), respectively. Chicken was moderate postively correlated with 2’-FL (*r*_*s*_ = 0.615). Pork meat consumption was weak positively correlated to LNFPI (*r*_*s*_ = 0.391) and total HMOs (*r*_*s*_ = 0.382), and moderate positively correlated to 2’-FL (*r*_*s*_ = 0.432).

In the fish group, tuna or sardine showed a weak positive correlation with the concentrations of LNnT (*r*_*s*_ = 0.374), whereas that some seafood showed a moderate positive correlation with LNT (*r*_*s*_ = 0.453).

Beans prepared at home, cooked, and refried showed a weak (*r*_*s*_ = 0.366) and moderate (*r*_*s*_ = 0.597) positive correlations with 3-FL concentrations, respectively).

In the cereals and tubers groups, whole grain bread and sweet bread both showed a moderate (*r*_*s*_ = 0.515) and weak (*r*_*s*_ = 0.342) positive correlation with 3-FL, respectively; whereas that potatoes (*r*_*s*_ = −0.389; half fried piece or half a potato pancake) showed a weak negative correlation and breakfast cereal (*r*_*s*_ = −0.912; fruit flavor; froot loops) showed a very strong negative correlation with 3-FL.

In the corn food, appetizers with vegetables (such as sopes, quesadillas, tlacoyos, gorditas, and enchiladas; not tacos) fried and without frying were moderate positively correlated with 3-FL (*r*_*s*_ = 0.509) and strong negatively correlated with 6’-SL (*r*_*s*_ = −0.660), respectively.

In the beverages group, the consumption of natural fruit waters with sugar was strong positively correlated with 3-FL (*r*_*s*_ = 0.708) and total HMOs (*r*_*s*_ = 0.664), unsweetened industrialized flavored beverages with 6’-SL (*r*_*s*_ = 0.673), and fruit nectars or fruit pulp industrialized with sugar were moderate positively associated with LNnT (*r*_*s*_ = 0.405) and 3’-SL (*r*_*s*_ = 0.443), while weak positively correlated to 6’-SL (*r*_*s*_ = 0.328) concentrations.

Soups with pasta (pasta soup) and tortilla (corn flour tortilla) groups were weak and strong positively correlated to 3’-SL (*r*_*s*_ = 0.369) and LNnT (*r*_*s*_ = 0.712) concentrations, respectively.

In the miscellaneous group, dried chili was strong and very strong positively associated with LNFPI (*r*_*s*_ = 0.7127) and 3-FL (*r*_*s*_ = 0.81742) concentrations, respectively; soy sauce, Worcestershire sauce, or liquid food seasonings were very strong positively associated with 3’-SL (*r*_*s*_ = 0.84515). The correlations between all food groups and HMOs are shown in Supplementary Table 4.

In addition, a statistical difference (p = 0.008) was observed in total daily calories between women with normal weight (4133.1 ± 1142.4 kCal) and women with overweight or obesity (3391.1 ± 1038.8 kCal). However, total daily calories and concentrations of each of the HMOs were not correlated.

## Discussion

The focus of the study was to describe the correlation between maternal factors and HMO concentration in colostrum samples. The absolute concentrations of LNFPI, 2’-FL, LNT, LNnT, 3’-SL, and 6’-SL were significantly lower in women with overweight or obesity compared to women with normal weight. In addition, from weak to moderate correlations between maternal age, previous births, prepregnancy weight, prepregnancy BMI, weight at delivery, and BMI at delivery, and HMOs concentrations at the beginning of lactation were observed. The frequency of consumption of food groups such as vegetables (poblano chili and 2’-FL; rs = 0.702, p = 0.0012), fruits (orange or tangerine and 3-FL; rs = 0.428, p = 0.0022) and meats (chicken and 2'-FL; rs = 0.615, p = 0.0039) was positively correlated to specific HMOs from moderate to strong correlations.

During pregnancy, many studies suggest that a medical history of diseases associated with lipid metabolism is linked to increased BMI^18^. Hernández-Higareda et al.^19^, conducted a study on 600 Mexican women from Guadalajara, Jalisco to identify pregnancy-related diseases associated with obesity. Like the present study, they observed a significant association between a medical history of cholesterolemia or dyslipidemia and overweight or obesity in the participating women. Although the size of the samplein the present study is limited, the average systolic blood pressure was also significantly higher in women with overweight or obesity. Studies in other countries and continents have previously reported this association^20–22^.

Being overweight or obese during pregnancy not only increases the risk of health complications in the woman but also affects the perinatal health of the newborn. Taoudi et al.^23^, studied 90 participants from Témara, Morocco, aged between 18 and 43 years and found a significant positive correlation between gestational BMI and newborn birth weight (*r*_*s*_ = 0.29; p < 0.001). A recent study in 1,112 Mexican women, reported that overweight marginally decreased the probability of having a low birth weight. In the present study, we observed that children born to women with overweight or obesity have a significant increase in birth weight compared to children born to women with normal weight^24,25^.

Approximately 15–20% of women worldwide do not express the *FUT2* gene and are considered non-secretors related to a lower diversity and concentration of fucosylated HMOs such as 2’-FL and LNFPI^26,27^. In the present study, we observed a low prevalence (5.7%) of the non-secretor state of mothers. The proportion of non-secretors and secretor women vary between each report and geographic location. A proportion of 0%, 32%, 36%, and 37% of non-secretors has been reported in Bolivia, USA, Gambia, and South Africa, respectively^28–31^. However, this can vary significantly in different regions within the same country due to the diversity of ethnic origins^15^.

Isganaitis et al.^12^, analyzed the relationships between maternal obesity and human milk metabolites in 31 mothers from Oklahoma, USA and found that LNFPI was reduced by about 62% in the breast milk of women with overweight or obesity compared to women with normal weight at one month of breastfeeding (p = 0.007). In the present study we observed a similar percentage reduction in LNFPI (63%) in the colostrum of women with overweight or obesity compared to women with normal weight although in the previous study their HMOs quantifications were relative by untargeted metabolomics. However, our results differ from the previous study in that no significant reduction in 2'-FL was observed compared to the 38% they observed^12^. Another study in 78 Brazilian mothers, observed that secretor women with overweight (BMI 25–29.9 kg m^−2^) had a significantly higher concentration of 2'-FL (p = 0.030) and lower 3'-FL concentration (p = 0.011) than secretor women with normal weight (BMI 18.5–24.9 kg m^−2^) about one month postpartum^32^. The results of that study, using a similar LC–MS analytical platform, were consistent with the present study in which 3-FL was found to be significantly reduced in women with overweight or obesity (p = 0.011). A fact to highlight is that the HMO content is highest in colostrum and tends to decrease over the course of lactation. These two studies were conducted in women at one month of lactation where 2’-FL can be reduced by close to 68% and 3-FL may be increased by around 215%.

In a study conducted by McGuire et al.^7^, maternal weight and BMI were negatively correlated with LNnT (*r*_*s*_ = −0.16 and *r*_*s*_ = −0.21, respectively) and positively correlated with 2′-fucosyllactose (*r*_*s*_ = 0.20 for both) in 410 healthy women from different countries at about three months postpartum. In addition, Samuel et al.^10^, found that women with overweight had significantly higher concentrations of 3′-SL (at day 2; colostrum), 6′-SL (at day 17) while lower concentrations of LNnT (at day 2; colostrum) and LNT (at day 30 and 90) compared to women with normal weight (p < 0.05 for all) in 370 women from seven European countries. These two reports were consistent with results found in the present study, where the concentrations of LNT and LNnT were lower in women with overweight or obesity compared to women with normal weight, although McGuire et al.^7^ used LC coupled to a duo ion-trap mass spectrometer for quantitative analysis. However, the increase of 3’-SL) concentration observed in women with overweight at day 2 of lactation by Samuel et al.^10^ disagrees with those found in the present study; LNnT was reduced in overweight women as was in the present study. Another study in 322 women from Brazil, prepregnancy BMI was moderate negatively correlated with 3-FL (*r*_*s*_ =  − 0.5), consistent with our study but discrepant in that prepregancy BMI was moderate positively correlated with LNnT (*r*_*s*_ = 0.4) at 2–8 days of lactation^11^.

The impact of other maternal factors (maternal age, mode of delivery, parity, and blood pressure) on the composition of HMOs remains unclear. Austin et al.^33^, conducted a cross-sectional study of 446 Chinese women in different stages of lactation (from 0–4 days to 4–8 months) and observed that the mode of delivery had no impact on HMOs composition. However, the study conducted by Samuel et al.^10^, observed that women giving birth through cesarean section (dystocic) had significantly higher concentrations of LNT (colostrum; at day 2) and 6′-SL (at day 30), and significantly lower concentrations of 2′-FL, 3′-SL, 6′-GL, LNFP III, LNnDFH and LNFP II (colostrum; at day 2) compared to those giving birth by vaginal delivery (eutocic). The present study is consistent only with those reported with Austin et al.^33^ in colostrum samples (< 48 h). Ferreira et al.^11^, reported that parity was moderate positively correlated with LNFP II (*r*_*s*_ = 0.4), DFLNT (*r*_*s*_ = 0.4), LNH (*r*_*s*_ = 0.4) and FDSLNH (*r*_*s*_ = 0.4) in colostrum samples at 2–8 days of lactation. Unlike the present study where parity showed a weak positive correlation with LNT and a weak negative correlation with 3'-SL, 6'-SL and LNnT.

McGuire et al.^7^, found that maternal age was weak negatively correlated with concentrations of LNnT, LSTc, and DSLNH (*r*_*s*_ =  − 0.14, *r*_*s*_ =  − 0.17, and *r*_*s*_ =  − 0.15, respectively) and was weak positively correlated with the concentration of FLNH (*r*_*s*_ = 0.15) from two weeks to five months of lactation. In the present study, maternal age was also weak negatively associated with LNnT concentrations at < 48 h of lactation.

In another studies, Fan et al.^15^, studied 468 pregnant women from Illinois USA and found that maternal age was weak positively correlated with relative abundances of 3-FL (*r*_*s*_ = 0.12, p = 0.019), DFLNHb (*r*_*s*_ = 0.11, p = 0.034), and IFLNH III (*r*_*s*_ = 0.1, p = 0.046), and weak negatively correlated with abundances of p-LNH (*r*_*s*_ =  − 0.13, p = 0.011) and IFLNH I (*r*_*s*_ =  − 0.13, p = 0.012) at 6 week of lactation. A study in 3,407 Canadian mothers conducted by Azad et al.^8^, lower concentrations of DFLNT and higher concentrations of LNnT and LNT in older mothers, while lower concentrations of 3-FL were observed in multiparous mothers and the mode of delivery was not associated with HMOs concentrations at 3–4 month of lactation^8^. In a longitudinal study of 116 Chinese mothers conducted by Wang et al.^34^, maternal age was weak negatively correlated with LNT + LNnT (*r*_*s*_ =  − 0.29), LNFPI (*r*_*s*_ =  − 0.21), but was weak positively correlated with 2′-FL (*r*_*s*_ = 0.22) only in secretor women at 1–5 days of lactation. In addition, they found a weak negative correlation between parity and LNFPI (*r*_*s*_ =  − 0.20) at 1–5 days of lactation. Tonon et al.^32^, observed a strong positive correlation between parity and LNT + LNnT (*r*_*s*_ = 0.79) and LNFPI (*r*_*s*_ = 0.79) levels and a strong negative correlation between parity and 3′-FL (*r*_*s*_ =  − 0.79) concentrations in secretor mothers (Se+) from 17 to 76 days of lactation.

Our results were consistent with the obtained by McGuire et al.^7^, and Wang et al.^34^, who observed a negative correlation between maternal age and LNnT concentrations. Regarding parity and composition of HMOs, our results are aligned with those obtained by Tonon et al.^32^, where parity showed a positive correlation with LNT concentrations. In addition, like Azad et al.^8^ and Samuel et al.^10^, no statistical differences were observed between the delivery mode and the concentrations of each of the HMOs evaluated in the present study.

Only a few studies investigated the impact of maternal diet on HMOs composition. Hallam et al.^35^, evaluated the effect of high-protein and high-prebiotic-fiber maternal diets on the composition of oligosaccharides in breast milk in maternal rats and observed an increase in both neutral and acidic oligosaccharides. Although the previous study is an animal model, our results show that the frequency of consumption of foods rich in fiber such as vegetables and legumes (beans) were positively correlated with the concentrations of HMOs.

In an interventional study using two different cohorts from Baylor, Texas tested the effect of glucose or galactose-enriched diet for 30–57 h (n = 7) or a high-fat or high-carbohydrate diets for 8 days (n = 7) on HMOs. HMO-bound fucose concentration was reduced with the glucose-enriched diet^16^. These results contrast with those found in the present study where we observed that the frequency of consumption of beverages rich in sugar increases the concentrations of fucosylated HMOs such as LNFPI and 3-FL.

Nutmeg is the richest in myristic acid (a food not typical in the Mexican diet), but we can find it in quantities 30–50 times lower in dairy products (such as yogurt), fish (such as tuna or sardines) and eggs^36^. This could explain the positive correlations observed in the present study between foods containing myristic acid and concentrations of LNT or LNnT. A source of stearic acid of plant origin is cereals (corn, rice, and others) which could be related to the findings of this study where negative correlations of 2'-FL and 6'-SL in consumers of breakfast cereal (fruit flavor) and appetizers with vegetables (such as sopes, quesadillas, tlacoyos, gorditas, and enchiladas; corn-based), respectively were observed.

Another study by Seppo et al.^37^, showed that supplementation with probiotics (*Lactobacillus rhamnosus* GG, *Lactobacillus rhamnosus* LC705, *Bifidobacterium breve* Bb99, and *Propionibacterium freudenreichii* subspecies *shermanii* JS) during the last three weeks of pregnancy increased the concentrations of 3-FL and 3′-SL in colostrum from 1223 pregnant mothers from Helsinki, Finland. In the present study, foods containing probiotics such as natural yogurt showed a positive correlation with LNnT and 6'-SL concentrations. However, the probiotic bacteria contained in this dairy food were not specified in the present study.

Fan et al.^15^, reported that higher cheese consumption was correlated with a higher amount of 2′-FL (p = 0.046) in breast milk at six weeks postpartum. In that same study, increased egg consumption was associated with greater LNT + LNnT abundance (p = 0.012). These results were contrary to those found in the present study, where the consumption of panela, fresh or cottage cheese, and semi or ripened cheeses showed a negative correlation with 3’-SL, 6’-SL, and LNnT concentrations. However, egg consumption was positively correlated with LNnT concentrations, consistent with what was reported by Fan et al.^15^. Quin et al.^38^, found that ingested carbohydrates (simple sugars and dietary fiber) were positively correlated with the relative levels of Galactose and Fucose present in HMOs with the decrease of Neu5Ac and Neu5Gc. Furthermore, they observed similar correlations for the total amount of fruit ingested, an important dietary source of sugars and fiber (a known source of Fucose). They observed significant positive correlations for Galactose and Fucose, while Neu5Ac levels were significantly lower in HMOs biosynthesized by women consuming large amounts of fruit. They observed that only cheese intake was positively associated with Neu5Gc levels and HMOs concentrations. These results are consistent with those found in the present study, showed that the consumption of different types of fruit was positively associated with the concentrations of all HMOs studied except 2'-FL.

Quin et al.^38^ demonstrated that Neu5Ac obtained from the diet from red meat and products derived from cow's milk (such as cheese) positively influences the concentrations of HMOs. Those above could explain the positive correlation observed in the present study between the consumption of red meat (pork meat and beef) and the concentrations of total HMOs.

Our study has some limitations, with the main restriction being the small sample size, which limits the power to detect differences between groups. Despite this, we found very revealing statistical differences consistent with those reported in the literature. In addition, the study was a single-center study, limiting the generalizability to other settings. However, regional studies where overweight and obesity are very prevalent among women and school-age children are necessary to improve our understanding of the influence of dietary habits, BMI, and other maternal factors on the composition of breast milk. The present study is the first to be carried out in Mexico, a country where about 30% of adults have obesity with a greater impact on women. The results of the present study show the impact of obesity on the composition of breast milk is significative, which would suggest consequences for the infant from birth.

In conclusion, the influence of overweight and obesity as a modifiable factor associated with lower concentrations of 2’-FL, LNFPI, 3’-SL, 6’-SL, LNT and LNnT at the beginning of lactation could affect the quality of breast milk and deprive the newborn of its associated health benefits. The frequency of consumption of certain food groups, such as fruits, vegetables, and meats, can increase the concentrations of specific HMOs. The present study opens the possibility of new research aimed to improve the quality of breast milk through nutritional intervention based on healthy eating and BMI control for overweight/obesity prevention.

## Methods

### Study design

The present was a cross-sectional, descriptive, prospective, and single-center study that was carried out at the Hospital Regional Materno Infantil de Alta Especialidad (HRMIAE) in Monterrey, Nuevo León, Mexico, over nine months from February 2023 to October 2023.

### Ethical approval

This study was conducted according to the ethical principles for medical research involving human subjects outlined in the Helsinki Declaration. Institutional review board approval (approval number: DEISC-PR-190122074; Hospital Regional Materno Infantil de Alta Especialidad) was obtained, and the participants provided written informed consent before participating in the study.

### Recruitment, eligibility criteria, and study groups

In the present study, 70 mothers from 18 to 49 years of age were recruited, and a signed written informed consent was previously obtained. Recruitment was in the rooming-in area (where recovering mothers and newborns stay together until their hospital discharge) at the HRMIAE, where the first contact was established with the participants. Then, the resident doctor or social service intern evaluated compliance with the following eligibility:

Inclusion criteria: (a) women with a full-term pregnancy (≥ 38 weeks); (b) Mexican nationality; (c) healthy; (d) 18 to 49 years of age; (e) that the weight and height record is in the pregnancy control record from the first trimester; (f) patient without a history of any diabetes.

Exclusion criteria: (a) history of antibiotic use in the three months before delivery; (b) prolonged exposure to antibiotics (> 3 weeks) during pregnancy; (c) immunosuppressive or immunomodulatory therapy with corticosteroids; (d) history of vegan, lacto-ovo-vegetarian or exclusion diet (for example ketogenic diet); (e) history of bariatric surgery; (f) exposure to antineoplastic drugs; (g) histamine-H2 receptor antagonists or proton pump inhibitors or monoclonal antibodies; (h) history of mental illness (those that make it impossible to take the sample); (i) history of alcohol, tobacco or drug use during pregnancy; j) neonates with fetal distress or contact with meconium; (k) neonates who were administered an antibiotic at birth; (l) patients who do not have reliable weight and height information during prenatal care; (m) patient with a history of metabolic diseases (gestational diabetes, diabetes mellitus type 1 and 2).

Elimination criteria: (a) antibiotics for more than 24 h after delivery; (b) intensive care (mother or baby) or any condition that prevents the collection of breast milk. (c) Patients who are unable for some reason to continue breastfeeding.

Mothers were categorized into two study groups (1: women with normal weight and 2: women with overweight or obesity) based on their pre-pregnancy BMI.The permanence in the study group until the conclusion of pregnancy was determined by total weight gain. This classification followed the 2009 IOM/NRC guidelines, which detail weight gain recommendations for women with singleton and twin fetuses in Table 2^39^. For example, women who had the diagnosis of normal weight (18.5–24.9 kg m^−2^), based on prepregnancy BMI and gained normal weight during pregnancy (11.3–15.9 kg) were classified as "women with normal weight", but if gained excessive weight during pregnancy was classified as "women with overweight or obesity." On the other hand, women who had the diagnosis of overweight (25.0–29.9 kg m^−2^) or obesity (≥ 30.0 kg m^−2^), based on prepregnancy BMI, but if gained normal weight during pregnancy (6.8–11.3 kg for overweight and 5–9 kg for obesity) remained classified as "women with overweight or obesity".

In addition, women were categorized according to their secretory status based on the ability to produce 2’-FL and LNFPI in breast milk.

### Data and sample collection

Socio-demographic data (age, residence, maximum level of studies, occupation), medical history, and anthropometric data were collected from all participants, and a food consumption frequency questionnaire was applied, which is a validated tool for the Mexican population within the nutritional evaluation that allows measuring caloric consumption and the portion consumed according to the frequency of consumption of food groups; total weekly frequency of food group consumption was calculated by multiplying the daily by the weekly frequency. Food consumption frequency data were collected at the same time as the colostrum sample collection and reflect the frequency in the last week (7 days).

In addition, relevant data associated with the delivery and newborn data were collected, such as anthropometrics, basic metabolic screening, and hearing screening routinely performed on all newborns. All information was collected using a *data collection instrument*.

About sample collection, 1 mL of breast milk samples (colostrum; < 48 h postpartum) were collected from all participating women with the help of medical personnel from the HRMIAE Human Milk Bank. After collection, the samples were immediately stored frozen at −20 °C. Finally, the samples were transported to the Centro de Biotecnología FEMSA of the Tecnologico de Monterrey and stored under the same freezing conditions until processing.

### Absolute quantification of human milk oligosaccharides

#### Sample preparation

The samples were prepared following the methodology previously validated in our laboratory by Urrutia Baca et al.^40^. 50 µL of each colostrum sample was diluted with 150 µL water and vortex mixed. Next, 50 µL of the mixture was transferred to 1.5 mL new tubes and mixed with 450 µL of water, and the mixture was centrifuged at 12,000×*g* for 30 min at 4 °C. Then, 300 µL of the lower aqueous phase was transferred to 1.5 mL tubes and mixed with 900 µL of acetonitrile. The mixture was sonicated for 10 min, incubated for 60 min at 4 °C, centrifuged at 12,000×*g* for 30 min at 4 °C, and the supernatant was recovered. For removal of residual protein, 500 µL of the supernatant was ultracentrifuged using tubes with a 3 kDa molecular weight cut-off membrane (Vivaspin 500, 3 kDa MWCO; GE28-9322-18; Sartorius, Stonehouse, UK) for 50 min at 7500×*g*, 4 °C. In this step, a carbohydrate fraction was obtained and filtered through a 0.22 μm nylon before UPLC–MS/MS analysis.

#### UPLC–MS/MS analysis

Calibration curves were made to establish the concentration ranges for quantification. For 2’-FL, 3-FL, and LNFPI, there were seven concentration points from 1 µg to 100 µg mL^−1^, and for 6’-SL, 3’-SL, LNT, and LNnT there were six concentration points from 1 to 50 µg mL^−1^. The linearity equations and the determination coefficient were obtained for both calibration curves.

The experiments were performed using an Acquity UPLC system (Waters, Milford, MA) equipped with Micromass Quattro Premier XE Mass Spectrometer (Waters, Milford, MA). An ACQUITY UPLC BEH Amide Column (Waters; 1.7 μm, 2.1 × 100 mm) coupled to an ACQUITY UPLC BEH Amide pre-column (Waters; 1.7 μm, 2.1 × 5 mm) was used for chromatographic separation adjusted at 40 °C using a binary gradient. The mobile phase A and B consisted of 10 mM ammonium formate in water and 99.9% acetonitrile, respectively. The following UPLC gradient was used: 0 − 2 min from 98 to 95% B, 2–3 min to 65% B, 3–8.5 min to 55% A, 8.5–11 min to 55% A, 11–11.2 min to 98% A, 11.2–14 min to 98% B. The flow rate was set to 0.4 mL min^−1^.

Mass spectrometric analyses were performed with a triple quadrupole spectrometer equipped with an electrospray ionization source (ESI). ESI conditions were as follows: Capillary 4 kV, Cone 50 V, Extractor 3 V, RF Lens 0 V, Source temperature 150 °C, desolvation temperature 300 °C, gas flow desolvation 720 L h^−1^, gas flow cone 300 L h^−1^. High-purity argon was used as collision gas. The mass spectrometer was operated in negative-ion, multiple reaction monitoring (MRM) mode. Our work team previously optimized MRM conditions (Supplementary Table 1) and carried out the validation of the method applied in the present study^40^.

### Statistical analysis

The distribution of data was determined for each study variable by Kolmogorov–Smirnov test. Next, associations were established between the mother and the newborn variables based on the study groups. Student’s t-test and Chi-square with Fisher’s Exact Test were used for continuous and categorical variables, respectively. The concentration values of each HMOs, total, and profile showed no normal distribution; therefore, a non-parametric Mann–Whitney U test was used to explore the differences between the study groups. The correlation between HMOs concentrations and factors including age, anthropometrics, previous births, and frequency of food group consumption were calculated by Spearman's correlation test; Spearman's correlation coefficient > 0 indicates a positive correlation and < 0 indicates a negative correlation. The maternal variables were organized into clusters on the X axis and the concentrations of each specific HMOs were organized on the Y axis by hierarchical cluster analysis based on ρ values. Finally, the total frequency of consumption of each food group was calculated and then correlated with the concentrations of each HMOs by Spearman's correlation test. For all statistical tests, a p-value less than 0.05 was considered significant. Finally, the box charts, heatmaps, and dendrograms were generated using Origin v2023.

### Supplementary Information

Supplementary Tables.

## Data Availability

The datasets generated during and/or analyzed during the current study are available from the corresponding author on reasonable request.
