# Supplementary material for: Exploring the impact of maternal factors and dietary habits on human milk oligosaccharide composition in early breastfeeding among Mexican women
Source: Sci Rep. 2024 Jun 26;14:14685. doi: 10.1038/s41598-024-63787-1 (PMC11199484; doi:10.1038/s41598-024-63787-1)
Supplement: Supplementary file 1 — Supplementary Tables. [file 41598_2024_63787_MOESM1_ESM.docx]

**Table S1.** Optimized mass spectrometry conditions for the seven analytical standards through Multiple Reaction Monitoring (MRM) using an electrospray ionization (ESI) in the negative ion mode.

| **Analyte** | **Molecular formula** | **Structure** | **Monoisotopic mass** | **Precursor Ion *(m z^-1^)* [M-H]^-^** | **Product Ion (*m z^-1^*)** | **Cone Energy (V)** | **Collision Energy (V)** |
| --- | --- | --- | --- | --- | --- | --- | --- |
| 2’-FL | C_18_H_32_O_15_ | Fucα1-2Galβ1-4Glc | 488.2 | 487.3 | 205.0 | 30 | 20 |
| 3-FL | C_18_H_32_O_15_ | Galβ1-4(Fucα1-3)Glc | 488.2 | 487.2 | 178.8 | 20 | 20 |
| 6’-SL | C_23_H_39_NO_19_ | Neu5Acα2-6Galβ1-4Glc | 633.2 | 632.3 | 290.1 | 50 | 30 |
| 3’-SL | C_23_H_39_NO_19_ | Neu5Acα2-3Galβ1-4Glc | 633.2 | 632.4 | 289.9 | 50 | 30 |
| LNT | C_26_H_45_NO_21_ | Galβ1–3GlcNAcβ1–3Galβ1–4Glc | 707.2 | 706.3 | 178.8 | 30 | 25 |
| LNnT | C_26_H_45_NO_21_ | Galβ1–4GlcNAcβ1–3Galβ1–4Glc | 707.2 | 706.2 | 142.9 | 30 | 32 |
| LNFPI | C_32_H_55_NO_25_ | Fucα1-2Galβ1-3GlcNAcβ1-3Galβ1-4Glc | 853.3 | 852.3 | 325.0 | 30 | 20 |

[M-H]^−^: deprotonated ions; Glc: glucose; Gal: galactose; GlcNAc: N-acetylglucosamine; Fuc: fucose; Neu5Ac: N-acetylneuraminic acid.

**Table S2.** Total, neutral, and acidic HMOs in colostrum samples from Mexican women with normal weight or overweight/obesity.

| **HMOs Profile**  **mean ± SD (%)** | **Concentration (g L^-1^)** | | | **P-value ^f^** |
| --- | --- | --- | --- | --- |
|  | **Total N=70** | **Normal weight n=36** | **Overweight/Obesity n=34** |  |
| Total HMOs^a^ | 13.9±4.7 (100) | 15.9±4.8 (100) | 11.7±3.4 (100) | <0.001 |
| Total Neutral^b^ | 11.7±4.3 (84.6) | 13.3±4.6 (83.8) | 10.0±3.2 (85.8) | 0.002 |
| Neutral Fucosylated^c^ | 8.8±3.3 (63.5) | 9.8±3.8 (61.7) | 7.7±2.2 (66.0) | 0.007 |
| Neutral No Fucosylated^d^ | 2.9±1.7 (21.1) | 3.5±1.6 (22.1) | 2.3±1.4 (19.7) | 0.003 |
| Total Acidic^e^ | 2.1±1.9 (15.4) | 2.6±1.9 (16.2) | 1.7±1.7 (14.2) | 0.010 |

a Mean ± Standard Deviation of the sum based on 2'-Fucosyllactose, 3-Fucosyllactose, Lacto-N-Tetraose, Lacto-N-neotetraose, Lacto-N-Fucopentaose I, 3'-Sialyllactose and 6'-Sialyllactose.

b Mean ± Standard Deviation of the sum based on 2'-Fucosyllactose, 3-Fucosyllactose, Lacto-N-Tetraose, Lacto-N-neotetraose and Lacto-N-Fucopentaose I.

c Mean ± Standard Deviation of the sum based on 2'-Fucosyllactose, 3-Fucosyllactose and Lacto-N-Fucopentaose I.

d Mean ± Standard Deviation of the sum based on Lacto-N-Tetraose and Lacto-N-neotetraose.

e Mean ± Standard Deviation of the sum based on 3'-Sialyllactose and 6'-Sialyllactose.

f The p values were obtained using the non-parametric Mann-Whitney U test.

**Table S3.** Comparison between total weekly food group consumption frequencies and study groups.

| **Food groups** | | **Total** | | **Women with normal weight** | | **Women with overweight or obesity** | | **p value ^a^** |
| --- | --- | --- | --- | --- | --- | --- | --- | --- |
|  |  | **Mean** | **SD** | **Mean** | **SD** | **Mean** | **SD** |  |
| I. Dairy foods | 1: Whole milk | 7.4 | 6.1 | 7.3 | 7.1 | 7.4 | 5.0 | 0.513 |
|  | 2: Skim milk | 5.4 | 2.3 | 5.4 | 2.2 | 5.4 | 2.6 | 1.0001 |
|  | 3: Natural whole yogurt | 5.5 | 3.5 | 4.3 | 3.3 | 7.5 | 3.3 | 0.2301 |
|  | 4: Whole yogurt with fruits | 5.0 | 5.0 | 5.0 | 5.9 | 5.0 | 2.4 | 0.4541 |
|  | 5: Low-fat yogurt with fruits or natural | 3.3 | 3.2 | 4.0 | 4.2 | 2.0 | 0.0 | 1.0001 |
|  | 6: Natural drinkable yogurt | 3.1 | 2.7 | 3.8 | 2.9 | 1.5 | 0.7 | 0.3811 |
|  | 7: Drinkable yogurt with fruits | 4.2 | 3.0 | 4.0 | 2.3 | 4.4 | 3.6 | 0.9011 |
|  | 8: Low-fat drinkable yogurt with fruits or natural | 2.0 | 1.2 | 1.7 | 1.2 | 3.0 | 0.0 | 0.5001 |
|  | 9: Panela, fresh or cottage cheese | 3.3 | 3.3 | 3.7 | 4.2 | 2.9 | 2.0 | 0.6671 |
|  | 10: Semi or ripened cheeses (Chihuahua, Gouda, Manchego) | 2.6 | 2.3 | 2.5 | 2.7 | 2.8 | 1.8 | 0.351 |
|  | 11: Danonino (Petit suisse) or similar | 4.4 | 4.7 | 3.0 | 2.8 | 5.8 | 6.2 | 0.6861 |
|  | 12: Yakult or similar | 2.8 | 3.6 | 3.2 | 4.0 | 1.3 | 0.6 | 0.4691 |
| II. Fruits | 1: Banana | 6.2 | 7.6 | 6.7 | 7.9 | 5.7 | 7.4 | 0.113 |
|  | 2: Fried plantain | 1.0 | 0.0 | 1.0 | 0.0 | 1.0 | 0.0 | 1.0001 |
|  | 3: Jicama | 1.5 | 0.7 | 1.7 | 0.8 | 1.2 | 0.4 | 0.0841 |
|  | 4: Orange or tangerine | 7.4 | 10.0 | 8.4 | 11.2 | 6.2 | 8.7 | 0.333 |
|  | 5: Apple or pear | 4.3 | 4.9 | 5.1 | 4.8 | 3.4 | 5.0 | **0.002** |
|  | 6: Melon or watermelon | 2.8 | 3.0 | 3.1 | 3.0 | 2.5 | 3.1 | 0.12 |
|  | 7: Guava | 5.0 | 9.6 | 2.7 | 4.0 | 7.6 | 13.2 | 0.6041 |
|  | 8: Mango | 4.3 | 7.1 | 6.0 | 9.2 | 2.3 | 3.0 | **0.007** |
|  | 9: Papaya | 3.3 | 4.1 | 3.8 | 4.1 | 2.6 | 4.0 | 0.0661 |
|  | 10: Pineapple | 2.7 | 4.6 | 3.3 | 5.0 | 2.3 | 4.3 | 0.4011 |
|  | 11: Pink grapefruit | 1.0 | 0.0 | 1.0 | 0.0 | 1.0 | 0.0 | 1.0001 |
|  | 12: Strawberries | 4.3 | 7.9 | 4.3 | 6.1 | 4.4 | 9.7 | 0.099 |
|  | 13: Grapes | 3.4 | 5.0 | 4.6 | 6.6 | 2.1 | 1.9 | 0.115 |
|  | 14: Peach | 2.1 | 2.1 | 4.0 | 3.0 | 1.2 | 0.4 | 0.1671 |
|  | 15: Fruits in syrup | 1.3 | 0.5 | 1.0 | 0.0 | 1.5 | 0.7 | 1.0001 |
|  | 16: Crystallized or dried fruits | 2.1 | 1.9 | 1.8 | 1.5 | 2.3 | 2.2 | 0.7881 |
| III. Vegetables | 1: Tomato | 7.1 | 7.7 | 6.7 | 6.3 | 7.5 | 9.3 | 0.729 |
|  | 2: Green leaves | 3.7 | 3.8 | 5.2 | 4.4 | 1.8 | 1.8 | **0.0021** |
|  | 3: Chayote squash | 2.3 | 3.2 | 3.1 | 4.5 | 1.6 | 1.8 | 0.2721 |
|  | 4: Carrot | 2.3 | 1.7 | 2.3 | 1.4 | 2.3 | 2.0 | 0.402 |
|  | 5: Pumpkin | 2.2 | 1.7 | 2.4 | 1.8 | 2.0 | 1.6 | 0.275 |
|  | 6: Broccoli | 2.2 | 1.8 | 2.8 | 2.1 | 1.8 | 1.5 | 0.046 |
|  | 7: Cabbage | 2.4 | 2.6 | 3.0 | 2.8 | 2.1 | 2.7 | 0.5031 |
|  | 8: Green beans | 1.2 | 0.6 | 1.7 | 1.2 | 1.0 | 0.0 | 0.5171 |
|  | 9: Corn | 1.9 | 1.6 | 2.0 | 1.5 | 1.9 | 1.7 | 0.571 |
|  | 10: Lettuce | 3.4 | 3.3 | 3.9 | 3.1 | 3.0 | 3.5 | 0.035 |
|  | 11: Nopales (cactus) | 2.3 | 2.5 | 2.9 | 3.1 | 1.7 | 1.4 | 0.09 |
|  | 12: Cucumber | 2.3 | 2.5 | 2.1 | 1.5 | 2.5 | 3.1 | 0.541 |
|  | 13: Avocado | 2.9 | 2.8 | 3.0 | 2.9 | 2.8 | 2.8 | 0.498 |
|  | 14: Poblano chili | 4.0 | 9.4 | 6.6 | 12.7 | 1.1 | 0.3 | 0.1131 |
|  | 15: Onion | 10.3 | 9.2 | 10.7 | 8.9 | 9.8 | 9.9 | 0.635 |
|  | 16: Packaged vegetables | 1.6 | 0.8 | 1.6 | 0.7 | 1.7 | 0.9 | 0.9051 |
|  | 17: Frozen vegetables | 1.8 | 1.0 | 1.9 | 0.7 | 1.6 | 1.3 | 0.3431 |
| IV. Meats, sausages, and eggs | 1: Pork meat | 1.8 | 0.9 | 1.9 | 0.8 | 1.7 | 1.0 | 0.193 |
|  | 2: Beef | 2.3 | 1.3 | 2.5 | 1.1 | 2.2 | 1.4 | 0.087 |
|  | 3: Dried beef | 2.0 | 1.7 | 4.0 | 0.0 | 1.0 | 0.0 | 0.6671 |
|  | 4: Sausage or chorizo | 2.3 | 2.0 | 2.2 | 1.1 | 2.5 | 2.7 | 0.73 |
|  | 5: Pork, turkey or combination sausage, pork or turkey ham or mortadella | 5.1 | 8.0 | 5.6 | 8.5 | 4.6 | 7.6 | **0.013** |
|  | 6: Chicken (1 piece leg/thigh or 1/2 piece small breast) | 2.2 | 1.4 | 2.6 | 1.7 | 1.8 | 0.8 | **0.033** |
|  | 7: Chicken (1 piece of wing, 2 pieces of leg) | 1.9 | 0.8 | 2.1 | 0.8 | 1.7 | 0.9 | 0.2091 |
|  | 8: Chicken (1 piece of liver or gizzard) | 2.4 | 1.0 | 2.2 | 0.8 | 3.0 | 1.4 | 0.5711 |
|  | 9: Egg (1 piece warm or boiled egg) | 7.0 | 8.1 | 7.1 | 5.9 | 6.8 | 10.7 | 0.1861 |
|  | 10: Egg (1 piece fried, fried or scrambled egg) | 6.3 | 7.0 | 7.3 | 6.9 | 5.3 | 7.2 | **0.039** |
| V. Fish | 1: Fresh fish | 2.3 | 3.0 | 3.1 | 4.0 | 1.4 | 1.0 | 0.1821 |
|  | 2: Dry fish | 2.0 | 0.0 | 2.0 | 0.0 | - | - | - |
|  | 3: Tuna and sardine | 1.6 | 2.2 | 2.3 | 3.3 | 1.1 | 0.3 | 0.1221 |
|  | 4: Some seafood | 1.3 | 0.8 | 1.5 | 1.0 | 1.0 | 0.0 | 0.3141 |
| VI. Legumes | 1: Beans prepared at home (cooked) | 5.8 | 6.0 | 5.8 | 6.1 | 5.8 | 6.0 | 0.992 |
|  | 2: Beans prepared at home (refried) | 5.6 | 5.3 | 5.8 | 5.1 | 5.4 | 5.8 | 0.5181 |
|  | 3: Packaged or canned beans (cooked) | 1.5 | 0.5 | 1.5 | 0.5 | - | - | - |
|  | 4: Packaged or canned beans (refried) | 2.8 | 2.0 | 2.6 | 2.1 | 4.0 | 0.0 | 0.4441 |
|  | 5: Lentil, chickpea, yellow bean or kidney bean | 2.1 | 1.6 | 2.0 | 1.4 | 2.5 | 2.3 | 0.7441 |
| VII. Cereals and tubers | 1: Stewed rice | 3.5 | 3.6 | 3.6 | 3.6 | 3.4 | 3.8 | 0.338 |
|  | 2: Flaked oats, natural or toasted amaranth | 3.0 | 3.2 | 3.4 | 3.6 | 2.4 | 2.4 | 0.3171 |
|  | 3: White bread | 2.6 | 3.7 | 3.0 | 4.5 | 2.2 | 2.8 | 0.534 |
|  | 4: Whole grain bread | 2.7 | 3.1 | 2.6 | 2.4 | 2.8 | 3.7 | 0.9231 |
|  | 5: Sweet bread (except donuts and churros) | 3.0 | 2.9 | 3.0 | 2.9 | 3.0 | 2.9 | 0.614 |
|  | 6: Bakery donuts and churros | 2.5 | 2.6 | 1.4 | 0.5 | 3.5 | 3.4 | 0.0781 |
|  | 7: Whole grain crackers | 2.7 | 2.4 | 3.3 | 2.9 | 1.8 | 1.0 | 0.6101 |
|  | 8: Pretzels | 3.6 | 6.5 | 4.4 | 8.4 | 2.8 | 3.5 | 0.8001 |
|  | 9: Potatoes: half a cooked piece | 2.3 | 2.4 | 2.4 | 2.7 | 2.2 | 1.9 | 0.817 |
|  | 10: Potatoes: half fried piece or half a potato pancake | 2.3 | 2.5 | 2.7 | 2.9 | 1.8 | 1.7 | 0.0771 |
|  | 11: Breakfast cereal: chocolate (chocozucaritas, chocokrispis) | 5.1 | 4.2 | 5.1 | 4.1 | 5.1 | 4.5 | 0.9651 |
|  | 12: Breakfast cereal: light-figure care (special K) | 3.3 | 2.9 | 1.0 | 0.0 | 4.0 | 3.0 | 0.5001 |
|  | 13: Breakfast cereal: sweetened flakes (zucaritas) | 3.8 | 3.3 | 3.8 | 2.7 | 3.7 | 3.9 | 0.6711 |
|  | 14: Breakfast cereal: basic (corn flakes, unflavored puffed rice) | 5.3 | 4.6 | 6.1 | 5.3 | 4.0 | 2.8 | 0.4321 |
|  | 15: Breakfast cereal: varieties (apple jack, honey smacks, corn pops) | 7.0 | 0.0 | 7.0 | 0.0 | - | - | - |
|  | 16: Breakfast cereal: fruit flavor (froot loops) | 8.5 | 7.8 | 10.0 | 7.7 | 1.0 | 0.0 | 0.3331 |
|  | 17: Breakfast cereal: fiber (all bran) | 1.5 | 0.7 | 1.5 | 0.7 | - | - | - |
|  | 18: Breakfast cereal: multi-ingredients (Extra) | 4.3 | 2.3 | 7.0 | 0.0 | 3.0 | 0.0 | 0.6671 |
| VIII. Corn based food | 1: Appetizers with vegetables such as sopes, quesadillas, tlacoyos, gorditas and enchiladas (not tacos; without frying) | 1.8 | 0.9 | 1.4 | 0.8 | 2.2 | 0.8 | 0.1491 |
|  | 2: Appetizers with vegetables such as sopes, quesadillas, tlacoyos, gorditas and enchiladas (not tacos; fried) | 1.2 | 0.5 | 1.1 | 0.3 | 1.5 | 0.8 | 0.3851 |
|  | 3: Appetizers with beef, pork, chicken, organ meats, etc. such as tacos, quesadillas, tlacoyos, enchiladas, gorditas (without frying); | 1.6 | 0.8 | 1.4 | 0.7 | 1.9 | 0.8 | 0.2371 |
|  | 4: Appetizers with beef, pork, chicken, organ meats, etc. such as tacos, quesadillas, tlacoyos, enchiladas, gorditas (fried) | 2.1 | 3.8 | 2.7 | 5.3 | 1.5 | 0.8 | 0.9051 |
|  | 5: Pozole (all types) | 1.0 | 0.0 | 1.0 | 0.0 | 1.0 | 0.0 | 1.0001 |
|  | 6: Tamal (all types) | 1.1 | 0.3 | 1.1 | 0.4 | 1.1 | 0.3 | 0.954 |
|  | 7: Corn atole: with water or pozol | 3.0 | 2.8 | 1.7 | 1.2 | 7.0 | 0.0 | 0.5001 |
|  | 8: Corn atole: with milk | 3.3 | 5.7 | 4.6 | 7.1 | 1.5 | 1.7 | 0.0191 |
| IX. Beverages | 1: Regular soft drink | 7.4 | 9.0 | 7.1 | 8.9 | 7.6 | 9.1 | 0.639 |
|  | 2: Diet soda | 6.5 | 0.7 | - | - | 6.5 | 0.7 | - |
|  | 3: Coffee without sugar | 4.5 | 3.5 | 2.0 | 0.0 | 7.0 | 0.0 | 1.0001 |
|  | 4: Coffee with sugar (1 tablespoon) | 4.2 | 2.6 | 3.4 | 2.4 | 5.7 | 2.2 | 0.0671 |
|  | 5: Coffee with milk | 5.0 | 2.5 | 4.5 | 2.7 | 5.3 | 2.4 | 0.6481 |
|  | 6: Coffee with cream substitute | 2.0 | 0.7 | 2.0 | 0.8 | 2.0 | 0.0 | 1.0001 |
|  | 7: Tea without sugar | 2.0 | 1.4 | 2.0 | 1.4 | - | - | - |
|  | 8: Tea with sugar | 2.9 | 2.1 | 2.1 | 0.9 | 3.9 | 2.6 | 0.2361 |
|  | 9: Natural juices without sugar | 3.1 | 2.4 | 4.0 | 3.5 | 2.4 | 1.1 | 0.9051 |
|  | 10: Natural juices with sugar | 4.4 | 5.3 | 6.0 | 6.1 | 1.4 | 0.5 | **0.0071** |
|  | 11: Natural fruit waters without sugar | 3.5 | 2.8 | 4.0 | 4.2 | 3.3 | 2.6 | 1.0001 |
|  | 12: Natural fruit waters with sugar | 5.7 | 6.7 | 5.7 | 6.0 | 5.8 | 8.6 | 0.6791 |
|  | 13: Unsweetened industrialized flavored beverages (including diet ones such as Clight, Be-light, etc.) | 7.4 | 12.4 | 5.0 | 2.6 | 8.4 | 15.0 | 0.5171 |
|  | 14: Industrialized flavored beverages with sugar (frutsi, bonafina.) | 2.0 | 1.4 | 2.5 | 2.1 | 1.5 | 0.7 | 0.6671 |
|  | 15: Fruit nectars or fruit pulp industrialized with sugar (boing, jumex) | 4.4 | 5.1 | 4.8 | 5.4 | 4.0 | 4.8 | 0.35 |
|  | 16: Water | 24.0 | 12.4 | 26.0 | 12.1 | 21.9 | 12.6 | 0.176 |
|  | 17: Alcoholic beverages | 2.0 | 0.0 | - | - | 2.0 | 0.0 | - |
| X. Soups and pasta | 1: Chicken, beef, or vegetable broth | 2.1 | 1.8 | 2.0 | 1.0 | 2.2 | 2.5 | 0.359 |
|  | 2: Soup with vegetables | 2.5 | 2.3 | 2.3 | 1.4 | 2.8 | 3.1 | 0.874 |
|  | 3: Pasta soup (broth) | 3.4 | 4.2 | 2.7 | 2.0 | 4.2 | 5.9 | 0.894 |
|  | 4: Pasta soup (dry soup) | 2.0 | 3.1 | 1.7 | 0.8 | 2.4 | 4.7 | 0.245 |
|  | 5: Cream of vegetables | 2.1 | 2.1 | 2.6 | 2.6 | 1.3 | 0.6 | 0.7861 |
|  | 6: Instant soups | 1.3 | 0.6 | 1.1 | 0.4 | 1.4 | 0.8 | 0.6131 |
| XI. Tortilla | 1: Nixtamal corn tortilla (homemade) | 9.8 | 9.4 | 8.3 | 11.0 | 14.0 | 0.0 | 1.0001 |
|  | 2: Corn flour tortilla (homemade) | 7.4 | 6.8 | 12.7 | 9.1 | 5.1 | 4.6 | 0.1831 |
|  | 3: Tortilla (purchased) or factory-made tortilla | 29.0 | 23.3 | 29.3 | 20.4 | 28.7 | 26.5 | 0.458 |
|  | 4: Wheat flour tortilla | 14.7 | 16.5 | 20.1 | 19.4 | 10.1 | 12.5 | 0.0581 |
| XII. Miscellaneous | 1: Lemon for example in salads, broths, or meats | 5.6 | 8.2 | 5.0 | 7.1 | 6.3 | 9.3 | 0.723 |
|  | 2: Onion, for example in sauces, or broths (ground or whole) | 10.5 | 8.3 | 10.9 | 7.9 | 10.0 | 9.0 | 0.7411 |
|  | 3: Fresh chiles, for example in sauces, tacos, stews (ground or whole) | 9.1 | 8.2 | 7.2 | 6.7 | 12.5 | 9.9 | 0.2381 |
|  | 4: Packaged or canned chilies, for example in sandwiches, cakes, stews | 2.1 | 1.6 | 2.5 | 1.6 | 1.7 | 1.6 | 0.1711 |
|  | 5: Dried chili, for example in sauces, tacos, stews (ground or whole) | 3.8 | 2.9 | 4.3 | 3.0 | 3.0 | 2.8 | 0.6101 |
|  | 6: Green tomato and tomato, for example in sauces, tacos, or stews (ground or whole) | 5.5 | 7.9 | 4.5 | 5.0 | 6.5 | 10.1 | 0.302 |
|  | 7: Sugar (apart from that added to drinks, milk, tea, coffee, fruit water) for example in strawberries or bananas with cream | 7.1 | 10.2 | 7.7 | 11.5 | 6.1 | 7.6 | 1.0001 |
|  | 8: Margarine | 1.7 | 2.0 | 2.2 | 2.7 | 1.0 | 0.0 | 0.7301 |
|  | 9: Butter | 1.8 | 1.8 | 2.6 | 2.4 | 1.2 | 0.5 | 0.1091 |
|  | 10: Mayonnaise | 2.6 | 3.0 | 2.1 | 1.7 | 3.0 | 3.8 | 0.974 |
|  | 11: Cream | 2.1 | 1.8 | 2.5 | 1.9 | 1.8 | 1.7 | 0.085 |
|  | 12: Vegetable shortening | 3.9 | 2.4 | 4.4 | 2.4 | 3.5 | 2.4 | 0.2861 |
|  | 13: Animal fat (pork, beef) | 2.0 | 1.4 | 1.0 | 0.0 | 3.0 | 0.0 | 1.0001 |
|  | 14: Salt or seasoning with salt added to your foods | 14.1 | 8.5 | 14.1 | 9.2 | 14.2 | 8.0 | 0.871 |
|  | 15: Ketchup | 2.5 | 4.3 | 1.4 | 0.7 | 3.1 | 5.4 | 0.7811 |
|  | 16: Spicy snack sauce | 2.8 | 2.4 | 3.1 | 2.6 | 2.5 | 2.1 | 0.4781 |
|  | 17: Soy sauce, Worcestershire sauce, or liquid food seasonings | 4.5 | 8.1 | 7.7 | 11.5 | 1.3 | 0.6 | 1.0001 |

^a^ The p values that had a significant difference (p <0.05) using the non-parametric Mann-Whitney U test are shown in red bold type.

**Table S4.** Spearman correlations of HMOs concentrations with total weekly food group consumption frequencies.

| **Food groups** | | **Spearman's correlation coefficient ^a^** | | | | | | | |
| --- | --- | --- | --- | --- | --- | --- | --- | --- | --- |
|  |  | **LNFPI** | **LNnT** | **LNT** | **3'-SL** | **6'-SL** | **3-FL** | **2'-FL** | **Total HMOs ^b^** |
| I. Dairy foods | 1: Whole milk | 0.06811 | 0.03709 | -0.04696 | 0.08167 | 0.031 | 0.03942 | -0.16787 | -0.08991 |
|  | 2: Skim milk | -0.56376 | 0.02056 | -0.61003 | 0.09596 | -0.08911 | -0.02742 | -0.21248 | -0.35642 |
|  | 3: Natural whole yogurt | -0.32412 | 0.1785 | **-0.62382** | 0.39928 | 0.22547 | -0.19259 | -0.41337 | -0.41337 |
|  | 4: Whole yogurt with fruits | 0.16033 | 0.38615 | **-0.54663** | 0.46518 | 0.27324 | -0.36131 | -0.13549 | 0.10613 |
|  | 5: Low-fat yogurt with fruits or natural | -- | -- | -- | -- | -- | -- | -- | -- |
|  | 6: Natural drinkable yogurt | 0.37796 | **0.75593** | -0.47673 | 0.56695 | **0.85042** | 0.18898 | -0.18898 | 0.56695 |
|  | 7: Drinkable yogurt with fruits | 0.07995 | -0.03052 | 0.04251 | 0.09209 | -0.07474 | -0.07561 | -0.14414 | 0.06659 |
|  | 8: Low-fat drinkable yogurt with fruits or natural | 0 | 0 | 0 | 0.44721 | 0.44721 | 0 | 0 | 0.44721 |
|  | 9: Panela, fresh or cottage cheese | -0.18707 | -0.22697 | 0.02271 | **-0.42071** | **-0.4035** | -0.09697 | -0.05354 | -0.23807 |
|  | 10: Semi or ripened cheeses (Chihuahua, Gouda, Manchego) | -0.00377 | **-0.32246** | -0.00248 | -0.30509 | **-0.44286** | -0.00821 | -0.18516 | **-0.3168** |
|  | 11: Danonino (Petit suisse) or similar | 0.26817 | 0.38311 | 0.42142 | 0.22986 | 0.2554 | 0.33203 | 0.22454 | 0.4725 |
|  | 12: Yakult or similar | -0.04494 | 0.20675 | -0.09888 | 0.12585 | 0.02697 | 0.28765 | 0.10352 | -0.01798 |
| II. Fruits | 1: Banana | 0.19134 | 0.17254 | 0.01578 | 0.1323 | 0.19678 | 0.17168 | 0.00201 | 0.09644 |
|  | 2: Fried plantain | -- | -- | -- | -- | -- | -- | -- | -- |
|  | 3: Jicama | 0.24462 | 0.10226 | -0.00442 | 0.16943 | 0.14434 | -0.10974 | 0.00942 | 0.08495 |
|  | 4: Orange or tangerine | **0.29424** | 0.07513 | 0.18463 | -0.03706 | 0.1155 | **0.42799** | 0.13421 | 0.22686 |
|  | 5: Apple or pear | 0.22394 | **0.30997** | -0.0521 | **0.36407** | **0.41123** | 0.07373 | -0.0396 | **0.28465** |
|  | 6: Melon or watermelon | 0.18641 | 0.14653 | -0.11189 | 0.15621 | 0.22647 | 0.22917 | -0.01113 | 0.25403 |
|  | 7: Guava | 0.06598 | -0.08518 | **-0.55149** | 0.12146 | 0.14197 | -0.22128 | -0.18992 | -0.15666 |
|  | 8: Mango | 0.20255 | **0.36246** | -0.04432 | **0.35287** | **0.35825** | 0.01331 | 0.08736 | 0.23087 |
|  | 9: Papaya | 0.11305 | 0.19839 | -0.02413 | -0.05231 | 0.21872 | 0.31462 | 0.10128 | 0.03902 |
|  | 10: Pineapple | -0.04198 | 0.09015 | -0.16458 | 0.26674 | 0.03245 | -0.14317 | -0.04128 | -0.0735 |
|  | 11: Pink grapefruit | -- | -- | -- | -- | -- | -- | -- | -- |
|  | 12: Strawberries | **0.32245** | **0.31592** | -0.01602 | 0.16696 | **0.34679** | 0.27019 | 0.16813 | **0.32634** |
|  | 13: Grapes | 0.15658 | 0.1909 | -0.02378 | 0.12766 | 0.19018 | **0.30732** | -0.13636 | 0.20854 |
|  | 14: Peach | 0.07921 | -0.24857 | -0.38616 | -0.13862 | 0.21783 | 0.33665 | 0.46371 | 0.23764 |
|  | 15: Fruits in syrup | -0.2582 | 0.2582 | 0.7746 | -0.7746 | -0.7746 | 0.7746 | -0.7746 | -0.7746 |
|  | 16: Crystallized or dried fruits | -0.09476 | 0.29482 | -0.11582 | 0.35799 | 0.35799 | **0.74758** | 0.01496 | 0.05265 |
| III. Vegetables | 1: Tomato | 0.04327 | -0.01332 | -0.03386 | 0.05222 | 0.05189 | 0.07875 | -0.03176 | -0.02812 |
|  | 2: Green leaves | 0.27819 | **0.45189** | 0.02692 | 0.30239 | 0.37507 | -0.06281 | 0.18744 | 0.30598 |
|  | 3: Chayote squash | 0.16652 | 0.4159 | 0.15137 | 0.19827 | 0.31353 | 0.04051 | 0.12093 | 0.18761 |
|  | 4: Carrot | 0.15355 | 0.14687 | 0.07573 | 0.20732 | 0.19513 | **0.2978** | 0.06482 | 0.16368 |
|  | 5: Pumpkin | 0.21807 | 0.18924 | 0.03984 | 0.18945 | 0.16396 | -0.01979 | -0.06496 | 0.06359 |
|  | 6: Broccoli | 0.27607 | 0.23574 | 0.14269 | 0.13439 | 0.14007 | -0.0148 | -0.01953 | 0.19144 |
|  | 7: Cabbage | 0.24024 | 0.30934 | 0.49091 | 0.12777 | 0.0269 | 0.11096 | -0.28433 | 0.08742 |
|  | 8: Green beans | 0.52223 | 0.40618 | 0.52223 | -0.40618 | 0.05803 | 0.29013 | 0.17408 | 0.40618 |
|  | 9: Corn | 0.1082 | 0.14362 | -0.04234 | 0.22514 | 0.1949 | -0.04204 | -0.17151 | 0.06088 |
|  | 10: Lettuce | **0.30035** | **0.29192** | 0.1188 | **0.27873** | **0.31185** | 0.08403 | -0.0057 | **0.26365** |
|  | 11: Nopales (cactus) | **0.31565** | **0.34986** | -0.01509 | **0.29447** | **0.35062** | 0.14428 | 0.08684 | 0.25991 |
|  | 12: Cucumber | 0.04788 | 0.17248 | -0.08943 | 0.17628 | 0.2144 | **0.37004** | -0.07262 | 0.15755 |
|  | 13: Avocado | **0.36604** | **0.38069** | 0.00653 | 0.19075 | 0.23835 | 0.09139 | 0.12024 | **0.30245** |
|  | 14: Poblano chili | 0.19331 | **0.47587** | -0.05751 | 0.33854 | **0.60388** | -0.03513 | **0.70204** | **0.46628** |
|  | 15: Onion | 0.08476 | 0.18362 | -0.08466 | 0.15505 | 0.20256 | 0.10882 | 0.09978 | 0.15536 |
|  | 16: Packaged vegetables | **-0.49793** | -0.08445 | -0.26037 | -0.11062 | -0.00395 | -0.19646 | -0.2755 | -0.16981 |
|  | 17: Frozen vegetables | 0.34662 | 0.11808 | 0.14881 | 0.34662 | 0.36566 | -0.18664 | 0 | 0.1714 |
| IV. Meats, sausages, and eggs | 1: Pork meat | **0.39121** | 0.26539 | 0.1628 | -0.08684 | 0.13598 | 0.23754 | **0.43203** | **0.38152** |
|  | 2: Beef | 0.23276 | 0.2049 | 0.11396 | -0.00769 | 0.03856 | 0.14706 | 0.23276 | **0.26869** |
|  | 3: Dried beef | 1 | 0 | 0.86603 | -0.86603 | -0.86603 | -0.86603 | 1 | 0.86603 |
|  | 4: Sausage or chorizo | -0.05793 | 0.13616 | -0.06843 | -0.04662 | 0.13373 | 0.07424 | -0.0708 | -0.03259 |
|  | 5: Pork, turkey or combination sausage, pork or turkey ham or mortadella | 0.12166 | 0.1943 | 0.05584 | 0.06539 | 0.17065 | **0.25556** | 0.02862 | 0.13487 |
|  | 6: Chicken (1 piece leg/thigh or 1/2 piece small breast) | 0.17498 | 0.14028 | 0.04593 | 0.04384 | 0.06021 | 0.09198 | 0.15318 | 0.13659 |
|  | 7: Chicken (1 piece of wing, 2 pieces of leg) | 0.09001 | -0.05428 | 0.25067 | -0.16045 | -0.11283 | 0.12137 | **0.61536** | 0.09941 |
|  | 8: Chicken (1 piece of liver or gizzard) | -0.28068 | -0.44909 | 0.28068 | -0.6175 | -0.43038 | 0.50522 | -0.30861 | -0.13098 |
|  | 9: Egg (1 piece warm or boiled egg) | 0.20027 | 0.19326 | -0.09665 | 0.04406 | 0.20427 | 0.22831 | 0.00803 | 0.12266 |
|  | 10: Egg (1 piece fried, fried or scrambled egg) | 0.10021 | **0.31046** | -0.03451 | 0.21844 | 0.24909 | 0.0509 | -0.10947 | 0.08731 |
| V. Fish | 1: Fresh fish | 0.32774 | 0.11493 | 0.21513 | -0.00688 | -0.23576 | -0.11886 | -0.18138 | 0.07073 |
|  | 2: Dry fish | -- | -- | -- | -- | -- | -- | -- | -- |
|  | 3: Tuna and sardine | 0.2432 | **0.37428** | 0.07883 | 0.14881 | 0.22254 | 0.06789 | 0.07025 | 0.20235 |
|  | 4: Some seafood | 0.35203 | 0.30499 | **0.45274** | -0.00533 | 0.07571 | 0.26553 | 0.15036 | 0.36364 |
| VI. Legumes | 1: Beans prepared at home (cooked) | 0.23097 | 0.17689 | 0.09582 | 0.22951 | 0.23948 | **0.36567** | -0.09036 | 0.14204 |
|  | 2: Beans prepared at home (refried) | 0.16503 | 0.24909 | 0.25078 | 0.10064 | 0.35 | **0.59701** | -0.02281 | 0.22732 |
|  | 3: Packaged or canned beans (cooked) | 0.48795 | 0.68313 | 0 | 0.68313 | 0.68313 | 0.68313 | -0.29277 | 0.48795 |
|  | 4: Packaged or canned beans (refried) | 0.25649 | 0.52154 | 0.00859 | 0.47879 | 0.58139 | 0.35054 | -0.27359 | 0.25649 |
|  | 5: Lentil, chickpea, yellow bean or kidney bean | -0.04868 | -0.05325 | 0.11449 | -0.20859 | -0.2152 | 0.03268 | 0.14588 | 0.03856 |
| VII. Cereals and tubers | 1: Stewed rice | 0.1829 | 0.15825 | 0.01122 | 0.09812 | 0.10453 | 0.13213 | 0.0015 | 0.13369 |
|  | 2: Flaked oats, natural or toasted amaranth | -0.14152 | 0.13121 | -0.20729 | 0.08923 | 0.0602 | -0.20882 | -0.11708 | -0.11801 |
|  | 3: White bread | -0.1252 | 0.02626 | -0.08976 | 0.14367 | 0.11767 | 0.25335 | -0.08391 | 0.00421 |
|  | 4: Whole grain bread | 0.16763 | 0.16838 | 0.06747 | 0.13008 | 0.17502 | **0.51533** | 0.0795 | 0.12032 |
|  | 5: Sweet bread (except donuts and churros) | 0.0602 | -0.03638 | 0.26517 | -0.10291 | -0.05828 | **0.34214** | 0.0945 | 0.05871 |
|  | 6: Bakery donuts and churros | 0.148 | 0.03082 | -0.15076 | 0.14896 | 0.01892 | 0.01028 | -0.16149 | -0.11487 |
|  | 7: Whole grain crackers | 0.22934 | 0.12369 | 0.21483 | -0.16275 | -0.16926 | 0.14973 | -0.38576 | 0.13671 |
|  | 8: Pretzels | 0.14807 | -0.01397 | 0.01246 | -0.03034 | -0.22462 | -0.09561 | -0.02416 | -0.08208 |
|  | 9: Potatoes: half a cooked piece | 0.01269 | -0.03672 | -0.01937 | 0.02023 | 0.07964 | 0.28014 | 0.14336 | 0.12615 |
|  | 10: Potatoes: half fried piece or half | -0.03491 | -0.06435 | 0.19959 | -0.05966 | -0.17898 | **-0.38864** | -0.02516 | -0.00587 |
|  | 11: Breakfast cereal: chocolate (chocozucaritas, chocokrispis) | -0.07732 | -0.40704 | -0.00162 | -0.16583 | -0.20783 | -0.23918 | -0.31843 | -0.40273 |
|  | 12: Breakfast cereal: light-figure care (special K) | 1 | -0.63246 | 0.31623 | -0.31623 | -0.31623 | 0.63246 | -1 | 0.21082 |
|  | 13: Breakfast cereal: sweetened flakes (zucaritas) | 0.22659 | 0.19619 | -0.08695 | 0.06653 | 0.10705 | 0.1832 | 0.21026 | 0.17066 |
|  | 14: Breakfast cereal: basic (corn flakes, unflavored puffed rice) | 0.13347 | 0.19355 | -0.01417 | 0.14635 | 0.00378 | 0.0236 | -0.22975 | 0.03399 |
|  | 15: Breakfast cereal: varieties (apple jack, honey smacks, corn pops) | -- | -- | -- | -- | -- | -- | -- | -- |
|  | 16: Breakfast cereal: fruit flavor (froot loops) | 0.58849 | 0.29424 | -0.19405 | 0.52964 | 0.29424 | **-0.91216** | 0.29424 | 0.50022 |
|  | 17: Breakfast cereal: fiber (all bran) | -- | -- | -- | -- | -- | -- | -- | -- |
|  | 18: Breakfast cereal: multi-ingredients (Extra | 0.86603 | 0.86603 | 0 | 0.86603 | 0.86603 | 0.86603 | 0.86603 | 0.86603 |
| VIII. Corn based food | 1: Appetizers with vegetables such as sopes, quesadillas, tlacoyos, gorditas and enchiladas (not tacos; without frying) | -0.16093 | -0.50071 | 0.03414 | -0.46657 | **-0.66003** | 0.2276 | -0.27247 | -0.07966 |
|  | 2: Appetizers with vegetables such as sopes, quesadillas, tlacoyos, gorditas and enchiladas (not tacos; fried) | -0.14907 | 0.03976 | -0.03023 | -0.00954 | 0.06203 | **0.50893** | 0.18558 | -0.10656 |
|  | 3: Appetizers with beef, pork, chicken, organ meats, etc. such as tacos, quesadillas, tlacoyos, enchiladas, gorditas (without frying); | 0.13228 | -0.06901 | -0.01899 | -0.08972 | -0.17369 | -0.02991 | -0.08512 | 0.10697 |
|  | 4: Appetizers with beef, pork, chicken, organ meats, etc. such as tacos, quesadillas, tlacoyos, enchiladas, gorditas (fried) | 0.26864 | 0.05316 | 0.01174 | -0.05169 | -0.16205 | 0.05464 | -0.22792 | -0.0385 |
|  | 5: Pozole (all types) | -- | -- | -- | -- | -- | -- | -- | -- |
|  | 6: Tamal (all types) | 0.12279 | 0.19632 | 0.07048 | 0.09564 | 0.06544 | 0.10571 | -0.0126 | 0.15101 |
|  | 7: Corn atole: with water or pozol | -0.63246 | -0.94868 | 0.21082 | -0.73786 | -0.73786 | 0.63246 | 0.86603 | -0.63246 |
|  | 8: Corn atole: with milk | -0.03996 | 0.18255 | 0.00391 | -0.07563 | 0.06831 | 0.15226 | 0.25923 | 0.02344 |
| IX. Beverages | 1: Regular soft drink | -0.11371 | 0.06898 | -0.2599 | 0.24152 | 0.13428 | -0.08465 | -0.16995 | -0.07472 |
|  | 2: Diet soda | -- | -- | -- | -- | -- | -- | -- | -- |
|  | 3: Coffee without sugar | -- | -- | -- | -- | -- | -- | -- | -- |
|  | 4: Coffee with sugar (1 tablespoon) | -0.05104 | -0.31281 | 0.15239 | -0.36551 | -0.28269 | 0.28953 | -0.07816 | -0.11841 |
|  | 5: Coffee with milk | -0.04363 | -0.0471 | -0.02945 | -0.02248 | 0.16912 | -0.13968 | 0.17793 | 0.07011 |
|  | 6: Coffee with cream substitute | -0.22361 | -0.67082 | 0.44721 | -0.44721 | -0.67082 | 0.67082 | -0.67082 | -0.67082 |
|  | 7: Tea without sugar | -- | -- | -- | -- | -- | -- | -- | -- |
|  | 8: Tea with sugar | -0.37422 | -0.16404 | -0.0651 | -0.01303 | -0.16925 | 0.24867 | -0.2857 | -0.26559 |
|  | 9: Natural juices without sugar | -0.29069 | -0.171 | -0.36489 | 0.0513 | -0.05985 | -0.37619 | 0.00855 | -0.28214 |
|  | 10: Natural juices with sugar | -0.26575 | 0.37705 | -0.159 | 0.39068 | 0.14537 | -0.14991 | -0.40475 | -0.17262 |
|  | 11: Natural fruit waters without sugar | 0.29424 | 0.61791 | -0.77621 | 0.61791 | 0.47079 | -0.61791 | 0.26482 | -0.02942 |
|  | 12: Natural fruit waters with sugar | 0.36517 | 0.2416 | 0.4998 | 0.38361 | 0.33935 | **0.7082** | 0.38361 | **0.66394** |
|  | 13: Unsweetened industrialized flavored beverages (including diet ones such as Clight, Be-light, etc.) | 0.3338 | 0.37133 | -0.42168 | 0.42168 | **0.67344** | 0.5098 | -0.3352 | 0.15734 |
|  | 14: Industrialized flavored beverages with sugar (frutsi, bonafina.) | 0.94868 | -0.21082 | 0.73786 | -0.73786 | 0.31623 | 0.73786 | 0.94868 | 0.94868 |
|  | 15: Fruit nectars or fruit pulp industrialized with sugar (boing, jumex) | 0.24944 | **0.4052** | -0.22567 | **0.44312** | **0.32831** | -0.1677 | -0.05066 | 0.16687 |
|  | 16: Water | 0.13744 | 0.22602 | -0.11794 | 0.19885 | 0.17068 | 0.14523 | -0.12853 | 0.10975 |
|  | 17: Alcoholic beverages | -- | -- | -- | -- | -- | -- | -- | -- |
| X. Soups and pasta | 1: Chicken, beef, or vegetable broth | 0.17475 | 0.15844 | 0.01837 | 0.16621 | 0.13625 | 0.0793 | -0.05134 | 0.14084 |
|  | 2: Soup with vegetables | -0.12362 | 0.1097 | -0.15748 | 0.07435 | -0.01844 | 0.07612 | -0.03929 | -0.09692 |
|  | 3: Pasta soup (broth) | 0.0262 | 0.23874 | -0.20742 | **0.36909** | 0.22832 | 0.16339 | -0.14587 | 0.00322 |
|  | 4: Pasta soup (dry soup) | -0.10328 | -0.03924 | 0.05157 | 0.02537 | 0 | 0.01051 | -0.17088 | -0.02843 |
|  | 5: Cream of vegetables | 0.39406 | 0.10911 | 0.60737 | -0.05455 | 0.13639 | 0.10911 | -0.07881 | 0.16366 |
|  | 6: Instant soups | 0.33248 | 0.24553 | -0.27097 | 0.17391 | 0.23785 | -0.18159 | -0.03371 | 0.1867 |
| XI. Tortilla | 1: Nixtamal corn tortilla (homemade) | -0.10541 | -0.21082 | -0.94868 | -0.21082 | -0.21082 | -0.10541 | 0.94868 | -0.10541 |
|  | 2: Corn flour tortilla (homemade) | 0.52025 | **0.71224** | 0.09909 | 0.21677 | 0.44593 | -0.11148 | 0.12387 | 0.48928 |
|  | 3: Tortilla (purchased) or factory-made tortilla | -0.02799 | 0.17414 | -0.2311 | 0.14511 | 0.16055 | 0.16455 | -0.17038 | -0.06394 |
|  | 4: Wheat flour tortilla | 0.25362 | 0.09343 | -0.0091 | -0.24887 | 0.0328 | 0.08654 | 0.11002 | 0.17942 |
| XII. Miscellaneous | 1: Lemon for example in salads, broths, or meats | -0.05344 | 0.005 | -0.15182 | -0.02911 | -0.081 | 0.17162 | -0.14401 | -0.08791 |
|  | 2: Onion, for example in sauces, or broths (ground or whole) | -0.08671 | -0.07008 | -0.04995 | -0.12336 | -0.03623 | -0.05659 | -0.02111 | -0.18894 |
|  | 3: Fresh chiles, for example in sauces, tacos, stews (ground or whole) | -0.34525 | -0.3152 | 0.20115 | -0.41153 | -0.21261 | 0.26504 | 0.15187 | -0.19152 |
|  | 4: Packaged or canned chilies, for example in sandwiches, cakes, stews | 0.11678 | -0.00124 | 0.30962 | -0.16796 | -0.044 | -0.00372 | 0.22238 | 0.2287 |
|  | 5: Dried chili, for example in sauces, tacos, stews (ground or whole) | **0.7127** | 0.4671 | 0.6228 | 0.07785 | 0.1557 | **0.81742** | 0.25717 | 0.50602 |
|  | 6: Green tomato and tomato, for example in sauces, tacos, or stews (ground or whole) | -0.12309 | -0.10226 | -0.12072 | -0.24594 | -0.24957 | 0.04506 | -0.11193 | -0.15595 |
|  | 7: Sugar (apart from that added to drinks, milk, tea, coffee, fruit water) for example in strawberries or bananas with cream | 0.11413 | -0.08703 | -0.0803 | -0.11301 | -0.22805 | -0.13404 | -0.02714 | -0.16298 |
|  | 8: Margarine | 0.27386 | 0.54772 | -0.13693 | 0.54772 | 0.54772 | 0.54772 | 0.54772 | 0.54772 |
|  | 9: Butter | 0.05405 | 0.15575 | 0.02269 | -0.11038 | -0.07761 | -0.05533 | -0.19875 | -0.02202 |
|  | 10: Mayonnaise | 0.1027 | 0.04434 | -0.01782 | -0.04858 | -0.02456 | 0.25478 | 0.13638 | 0.09401 |
|  | 11: Cream | 0.22268 | 0.26128 | 0.16073 | -0.02139 | 0.17611 | 0.12281 | 0.04625 | 0.16978 |
|  | 12: Vegetable shortening | 0.34145 | 0.25672 | -0.02579 | 0.07623 | 0.01075 | -0.21474 | 0.10503 | 0.04965 |
|  | 13: Animal fat (pork, beef) | -- | -- | -- | -- | -- | -- | -- | -- |
|  | 14: Salt or seasoning with salt added to your foods | 0.0505 | 0.09865 | -0.12275 | 0.0299 | 0.11899 | 0.10862 | -0.02681 | 0.03482 |
|  | 15: Ketchup | 0.11476 | 0.19768 | -0.31658 | 0.15982 | 0.20643 | 0.11196 | -0.18664 | 0.07464 |
|  | 16: Spicy snack sauce | 0.23128 | 0.16475 | 0.05172 | 0.07561 | 0.18267 | 0.07426 | 0.05242 | 0.11631 |
|  | 17: Soy sauce, Worcestershire sauce, or liquid food seasonings | 0.35355 | 0.77754 | -0.43948 | **0.84515** | 0.77754 | -0.16903 | -0.35355 | 0.27045 |

^a^ Spearman's correlation coefficients that had a significant correlation (p <0.05) are shown in red bold type. Spearman's correlation coefficient >0 indicate a positive correlation and <0 indicate a negative correlation.

^b^ The sum is based on 2'-FL, 3-FL, LNT, LNnT, LNFPI, 3'-SL and 6'-SL.
